# Supplementary material for: Systematic review and meta-analysis of the evidence for an illusory truth effect and its determinants
Source: Nat Commun. 2026 Feb 27;17:3270. doi: 10.1038/s41467-026-70041-x (PMC13066098; doi:10.1038/s41467-026-70041-x)
Supplement: Supplementary file 1 — Supplementary Information [file 41467_2026_70041_MOESM1_ESM.pdf]

Supplementary Information

to **Systematic review and meta-Analysis of the Evidence for an Illusory Truth Effect and its Determinants,** by Steeven Ye, David Attali, Maria Ghazi, Arnaud Cachia, Mathieu Cassotti, & Grégoire Borst.

Table of Content

TABLE S1. PREFERRED REPORTING ITEMS FOR SYSTEMATIC REVIEWS AND META-ANALYSES (PRISMA) CHECKLIST[1]. 2

TABLE S2. SENSITIVITY ANALYSIS OF STANDARDIZED MEAN DIFFERENCE COMPUTATION BETWEEN BORENSTEIN'S AND BECKER'S FORMULA. 5

TABLE S3. EFFECT SIZES FOR THE ILLUSORY TRUTH EFFECT BY CUE TYPES AND VALENCE. 6

FIGURE S1-S16. FOREST PLOTS STRATIFIED BY KEY MODERATORS. 7

SUPPLEMENTARY METHODS 30

SUPPLEMENTARY REFERENCES 33

Table S1. Preferred Reporting Items for Systematic reviews and Meta-Analyses (PRISMA) checklist[1].

This checklist is reproduced from the PRISMA 2020 Checklist (Page et al., 2021). The PRISMA 2020 checklist is published under the Creative Commons Attribution 4.0 International (CC BY 4.0) license (https://creativecommons.org/licenses/by/4.0/).

Section and Topic	Item #	Checklist item	Location where item is reported
**TITLE**			
Title	1	Identify the report as a systematic review.	Title
**ABSTRACT**			
Abstract	2	See the PRISMA 2020 for Abstracts checklist.	Abstract
**INTRODUCTION**			
Rationale	3	Describe the rationale for the review in the context of existing knowledge.	Introduction
Objectives	4	Provide an explicit statement of the objective(s) or question(s) the review addresses.	L99-116
**METHODS**			METHODS
Eligibility criteria	5	Specify the inclusion and exclusion criteria for the review and how studies were grouped for the syntheses.	Eligibility Criteria
Information sources	6	Specify all databases, registers, websites, organisations, reference lists and other sources searched or consulted to identify studies. Specify the date when each source was last searched or consulted.	Information Sources, Search Strategy and Selection Process
Search strategy	7	Present the full search strategies for all databases, registers and websites, including any filters and limits used.	
Selection process	8	Specify the methods used to decide whether a study met the inclusion criteria of the review, including how many reviewers screened each record and each report retrieved, whether they worked independently, and if applicable, details of automation tools used in the process.	
Data collection process	9	Specify the methods used to collect data from reports, including how many reviewers collected data from each report, whether they worked independently, any processes for obtaining or confirming data from study investigators, and if applicable, details of automation tools used in the process.	Data Extraction and Coding
Data items	10a	List and define all outcomes for which data were sought. Specify whether all results that were compatible with each outcome domain in each study were sought (e.g. for all measures, time points, analyses), and if not, the methods used to decide which results to collect.	Effect Size Calculation and Missing Outcome of Interest + Data Extraction and Coding
	10b	List and define all other variables for which data were sought (e.g. participant and intervention characteristics, funding sources). Describe any assumptions made about any missing or unclear information.	Extraction of Moderating Variables
Study risk of bias assessment	11	Specify the methods used to assess risk of bias in the included studies, including details of the tool(s) used, how many reviewers assessed each study and whether they worked independently, and if applicable, details of automation tools used in the process.	Data Extraction and Coding + Risk of Bias
Effect measures	12	Specify for each outcome the effect measure(s) (e.g. risk ratio, mean difference) used in the synthesis or presentation of results.	Effect Size calculation and Missing Outcome of Interest

Section and Topic	Item #	Checklist item	Location where item is reported
Synthesis methods	13a	Describe the processes used to decide which studies were eligible for each synthesis (e.g. tabulating the study intervention characteristics and comparing against the planned groups for each synthesis (item #5)).	Data Extraction and Coding
	13b	Describe any methods required to prepare the data for presentation or synthesis, such as handling of missing summary statistics, or data conversions.	Effect Size Calculation + Missing Outcomes of Interest and Sensitivity Analyses
	13c	Describe any methods used to tabulate or visually display results of individual studies and syntheses.	Results + Transparency and Openness
	13d	Describe any methods used to synthesize results and provide a rationale for the choice(s). If meta-analysis was performed, describe the model(s), method(s) to identify the presence and extent of statistical heterogeneity, and software package(s) used.	Statistical Analyses
	13e	Describe any methods used to explore possible causes of heterogeneity among study results (e.g. subgroup analysis, meta-regression).	Extraction of Moderating Variables
	13f	Describe any sensitivity analyses conducted to assess robustness of the synthesized results.	Overall Effect Sizes and Sensitivity Analyses + Missing Outcome of Interest
Reporting bias assessment	14	Describe any methods used to assess risk of bias due to missing results in a synthesis (arising from reporting biases).	Publication Bias
Certainty assessment	15	Describe any methods used to assess certainty (or confidence) in the body of evidence for an outcome.	Missing Outcome of Interest and Sensitivity Analyses
**RESULTS**			
Study selection	16a	Describe the results of the search and selection process, from the number of records identified in the search to the number of studies included in the review, ideally using a flow diagram.	Study Selection and Study Characteristics + Figure 4
	16b	Cite studies that might appear to meet the inclusion criteria, but which were excluded, and explain why they were excluded.	Supplementary Data 5
Study characteristics	17	Cite each included study and present its characteristics.	Study Selection and Study Characteristics + Supplementary Data 1 + Supplementary References
Risk of bias in studies	18	Present assessments of risk of bias for each included study.	Risk of Bias
Results of individual studies	19	For all outcomes, present, for each study: (a) summary statistics for each group (where appropriate) and (b) an effect estimate and its precision (e.g. confidence/credible interval), ideally using structured tables or plots.	Overall Effect Size and Sensitivity Analysis + Table 1 + Supplementary Data 4
Results of	20a	For each synthesis, briefly summarise the characteristics and risk of bias among contributing studies.	Supplementary Data 2

Section and Topic	Item #	Checklist item	Location where item is reported
syntheses	20b	Present results of all statistical syntheses conducted. If meta-analysis was done, present for each the summary estimate and its precision (e.g. confidence/credible interval) and measures of statistical heterogeneity. If comparing groups, describe the direction of the effect.	Overall Effect Size and Sensitivity Analysis
	20c	Present results of all investigations of possible causes of heterogeneity among study results.	Meta-regression
	20d	Present results of all sensitivity analyses conducted to assess the robustness of the synthesized results.	Overall Effect Size and Sensitivity Analysis + Supplementary Data 3
Reporting biases	21	Present assessments of risk of bias due to missing results (arising from reporting biases) for each synthesis assessed.	Publication Bias
Certainty of evidence	22	Present assessments of certainty (or confidence) in the body of evidence for each outcome assessed.	Overall Effect Size and Sensitivity Analysis
**DISCUSSION**			
Discussion	23a	Provide a general interpretation of the results in the context of other evidence.	Discussion L380-521
	23b	Discuss any limitations of the evidence included in the review.	Discusssion L546-562
	23c	Discuss any limitations of the review processes used.	Discussion L522-545
	23d	Discuss implications of the results for practice, policy, and future research.	Discussion L563-576
**OTHER INFORMATION**			
Registration and protocol	24a	Provide registration information for the review, including register name and registration number, or state that the review was not registered.	Transparency and Openness
	24b	Indicate where the review protocol can be accessed, or state that a protocol was not prepared.	Transparency and Openness
	24c	Describe and explain any amendments to information provided at registration or in the protocol.	Not applicable
Support	25	Describe sources of financial or non-financial support for the review, and the role of the funders or sponsors in the review.	Acknowledgements
Competing interests	26	Declare any competing interests of review authors.	Competing interest statement
Availability of data, code and other materials	27	Report which of the following are publicly available and where they can be found: template data collection forms; data extracted from included studies; data used for all analyses; analytic code; any other materials used in the review.	Data availability + Code availability

From: Page MJ, McKenzie JE, Bossuyt PM, Boutron I, Hoffmann TC, Mulrow CD, et al. The PRISMA 2020 statement: an updated guideline for reporting systematic reviews. BMJ 2021;372:n71. doi: 10.1136/bmj.n71.
This work is licensed under CC BY 4.0. To view a copy of this license, visit https://creativecommons.org/licenses/by/4.0/

Table S2. Sensitivity analysis of standardized mean difference computation between Borenstein's and Becker's formula.

	Imputation of missing outcomes	*g*	SE	95% CI	Heterogeneity test ($Q(365)$)	τ^2 within	τ^2 between
Boreinstein's formula	Median	0.57	0.03	[0.51, 0.64]	5348	0.095	0.166
	Minimum	0.65	0.04	[0.57, 0.72]	4750	0.134	0.148
	Maximum	0.46	0.03	[0.40, 0.51]	26960	0.068	0.158
Becker's formula	Median	0.64	0.04	[0.56, 0.72]	6478	0.127	0.261
	Minimum	0.75	0.05	[0.66, 0.85]	6696	0.199	0.357
	Maximum	0.56	0.04	[0.49, 0.64]	14272	0.112	0.227

Effect sizes are reported as Hedges' g with 95% confidence intervals. Effect size estimates were pooled using the restricted maximum-likelihood (REML) estimators. Statistical inference was based on two-sided t tests with Knapp–Hartung adjustment. All P values are two-sided and no adjustment for multiple comparisons was applied. Heterogeneity statistics (τ^2, I^2) are reported at within- and between-study levels. See Supplementary Note 1 for details on the computation of effect sizes using Becker's formula. SE, standard error.

Table S3. Effect sizes for the illusory truth effect by cue types and valence.

The table reports the number of effect sizes, PEESE-corrected estimates, and 95% confidence intervals by type and valence of cues.

Category		Number of effect sizes (u)	Effect size (g)	95% CI
No cue		287	0.43	[0.36, 0.50]
Epistemic qualifiers	True cue	7	0.16	[-0.21, 0.52]
	False cue	10	0.01	[-0.27, 0.29]
Source reliability	True cue	16	0.64	[0.40, 0.87]
	False cue	16	-0.21	[-0.44, 0.02]
Labels	True cue	1	-0.60	[-2.25, 1.06]
	False cue	5	0.27	[-0.03, 0.57]
Immediate feedback	True cue	2	0.67	[0.30, 1.44]
	False cue	2	-0.58	[-1.12, -0.04]
Delayed feedback	True cue	7	0.75	[0.33, 1.17]
	False cue	7	-0.90	[-1.31, -0.50]

Figure S1-S16. Forest Plots Stratified by Key Moderators.

The following forest plots are stratified by key moderators: item type, exposure task, veracity cues, and presentation time during the exposure phase.

Figures S1–S4 display the PEESE-corrected bivariate overall effect sizes for each subgroup, along with their corresponding 95% confidence intervals.

Figures S5–S16 present the individual effect sizes and weights for each study, grouped by moderator levels. For readability, Figures S6, S9, S12 and S15 are presented in multiple panels, with each panel displayed on a separate page. Together, panels represent the complete set of study-level effect sizes for the corresponding moderator. These plots were generated using the first imputed dataset from the multiple imputation procedure. This approach was selected for clarity of visualization, as standard plotting functions do not support pooling forest plots across multiple imputations.

Given the consistency of pseudo-R^2 values across imputations (e.g., ranging from 0.35 to 0.41) and the robustness of the pooled estimates, the use of a single imputed dataset for visualization purposes should not substantially affect the interpretation of subgroup patterns.

All data and analysis code are publicly available via the project's OSF page, allowing to reproduce these visualizations using any of the imputed datasets.

Figure S1. PEESE-corrected effect sizes by item type. This forest plot displays PEESE-corrected bivariate overall effect sizes stratified by item type, with 95% confidence intervals. Estimates are based on the first imputed dataset and shown for visual comparison across subgroups.

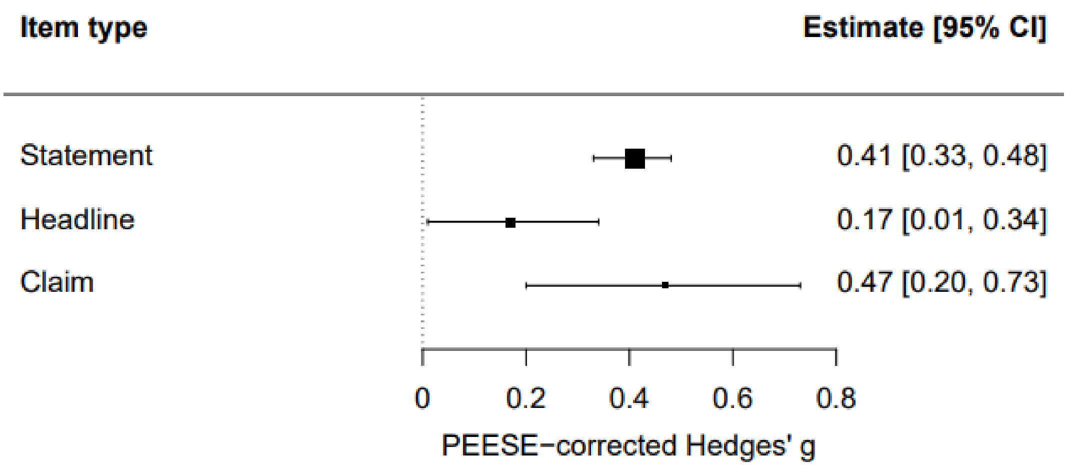

Figure S2. PEESE-corrected effect sizes by exposure task. This forest plot displays PEESE-corrected bivariate overall effect sizes stratified by exposure task, with 95% confidence intervals. Estimates are based on the first imputed dataset and shown for visual comparison across subgroups.

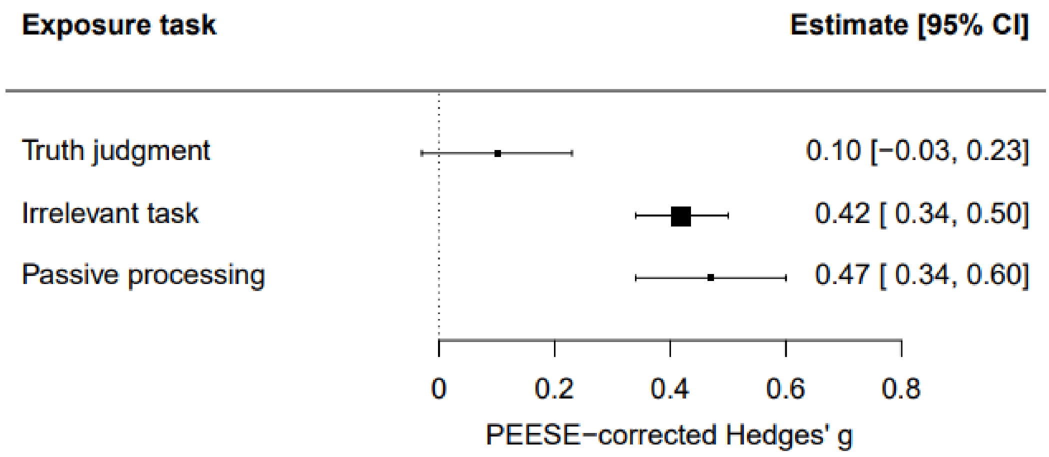

Figure S3. PEESE-corrected effect sizes by veracity cues. This forest plot displays PEESE-corrected bivariate overall effect sizes stratified by veracity cues, with 95% confidence intervals. Estimates are based on the first imputed dataset and shown for visual comparison across subgroups.

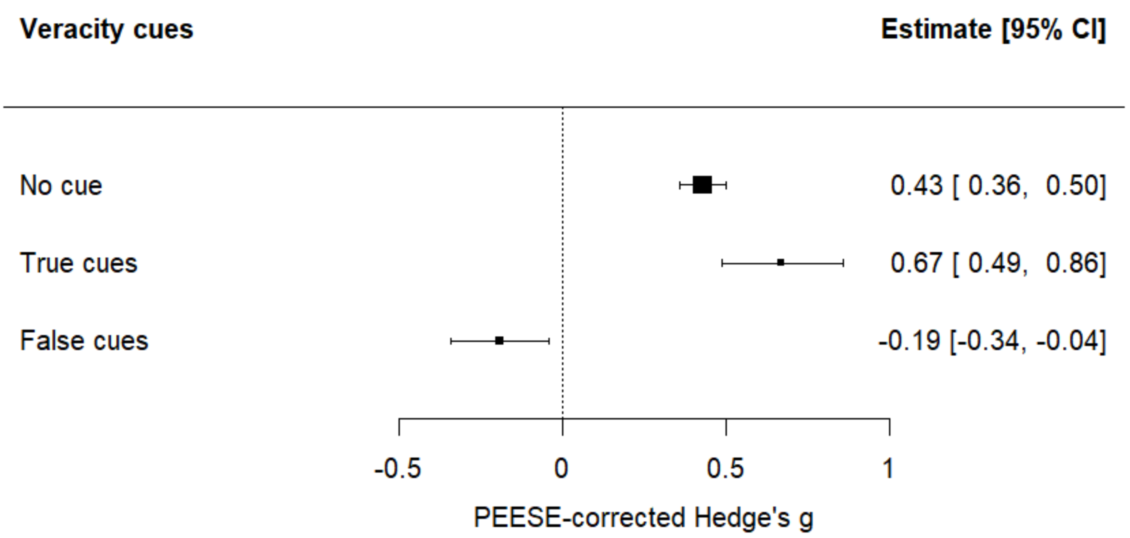

Figure S4. PEESE-corrected effect sizes by exposure time. This forest plot displays PEESE-corrected bivariate overall effect sizes stratified by exposure time, with 95% confidence intervals. Estimates are based on the first imputed dataset and shown for visual comparison across subgroups.

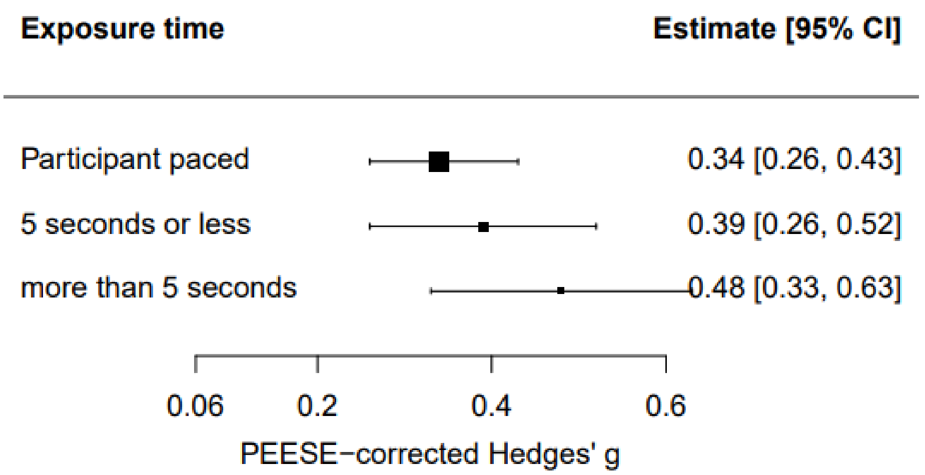

Figure S5. Forest plot by Item type: Claims

This forest plot shows individual study effect sizes (Hedges' g) with corresponding 95% confidence intervals for claim-based items. Effect sizes are based on the first imputed dataset and are displayed for visual comparison across studies.

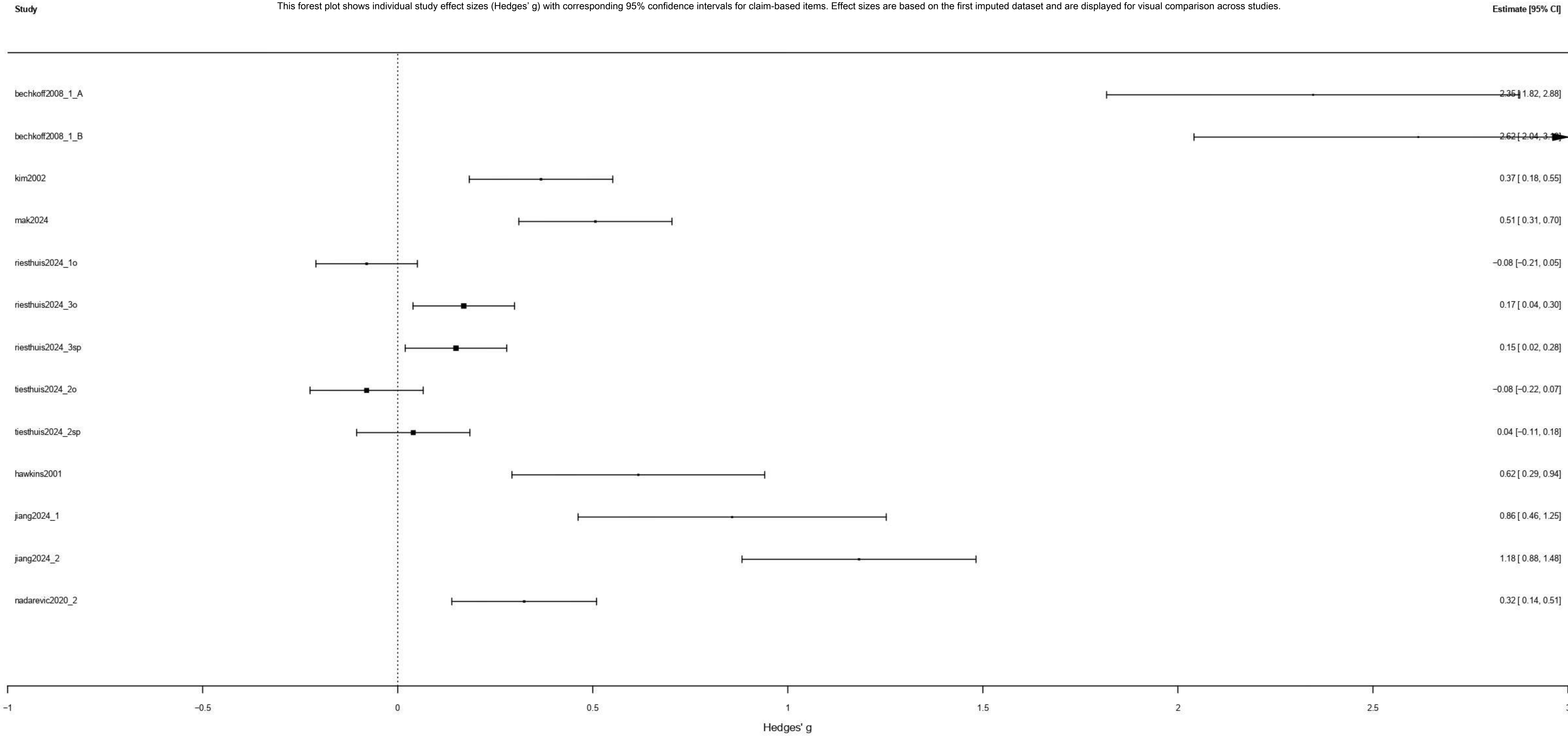

Figure S6a. Forest plot by Item type: Statements

This forest plot shows the first group of individual study effect sizes (Hedges' g) with corresponding 95% confidence intervals for statements-based items. Effect sizes are based on the first imputed dataset and are displayed for visual comparison across studies.

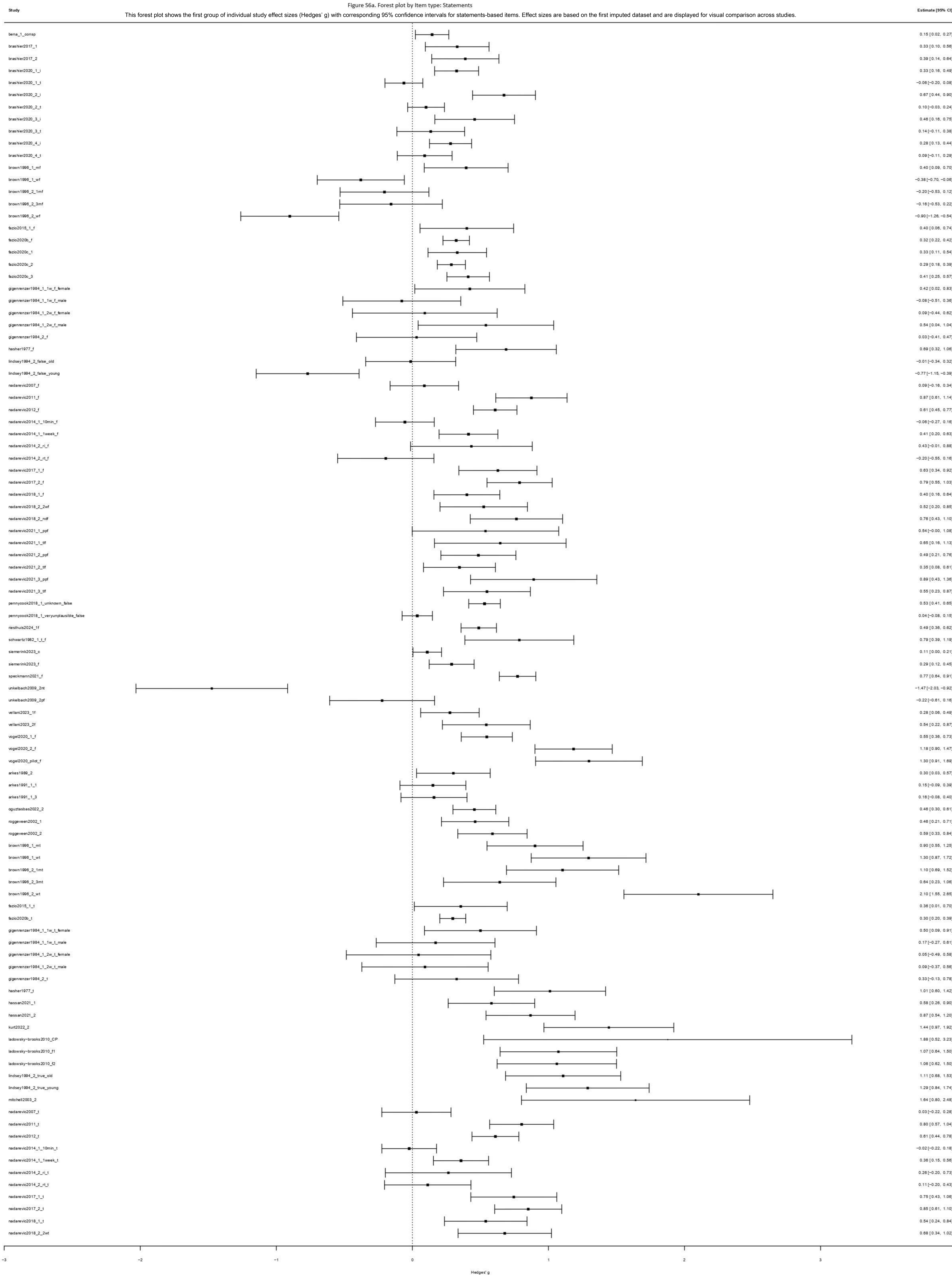

Figure S6b. Forest plot by Item type: Statements

This forest plot shows the second group of individual study effect sizes (Hedges' g) with corresponding 95% confidence intervals for statements-based items. Effect sizes are based on the first imputed dataset and are displayed for visual comparison across studies.

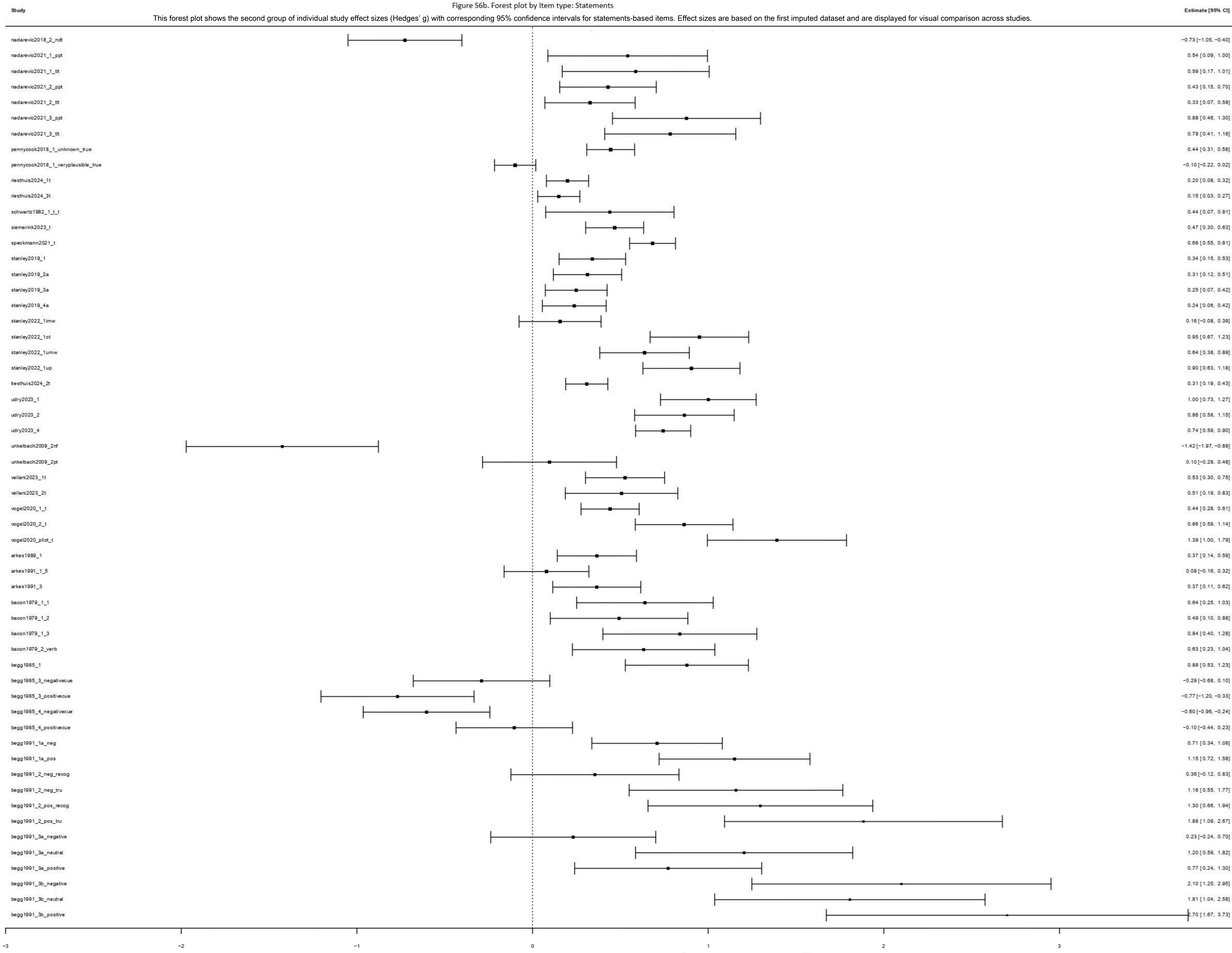

Figure S6c. Forest plot by Item type: Statements

This forest plot shows the third group of individual study effect sizes (Hedges' g) with corresponding 95% confidence intervals for statements-based items. Effect sizes are based on the first imputed dataset and are displayed for visual comparison across studies.

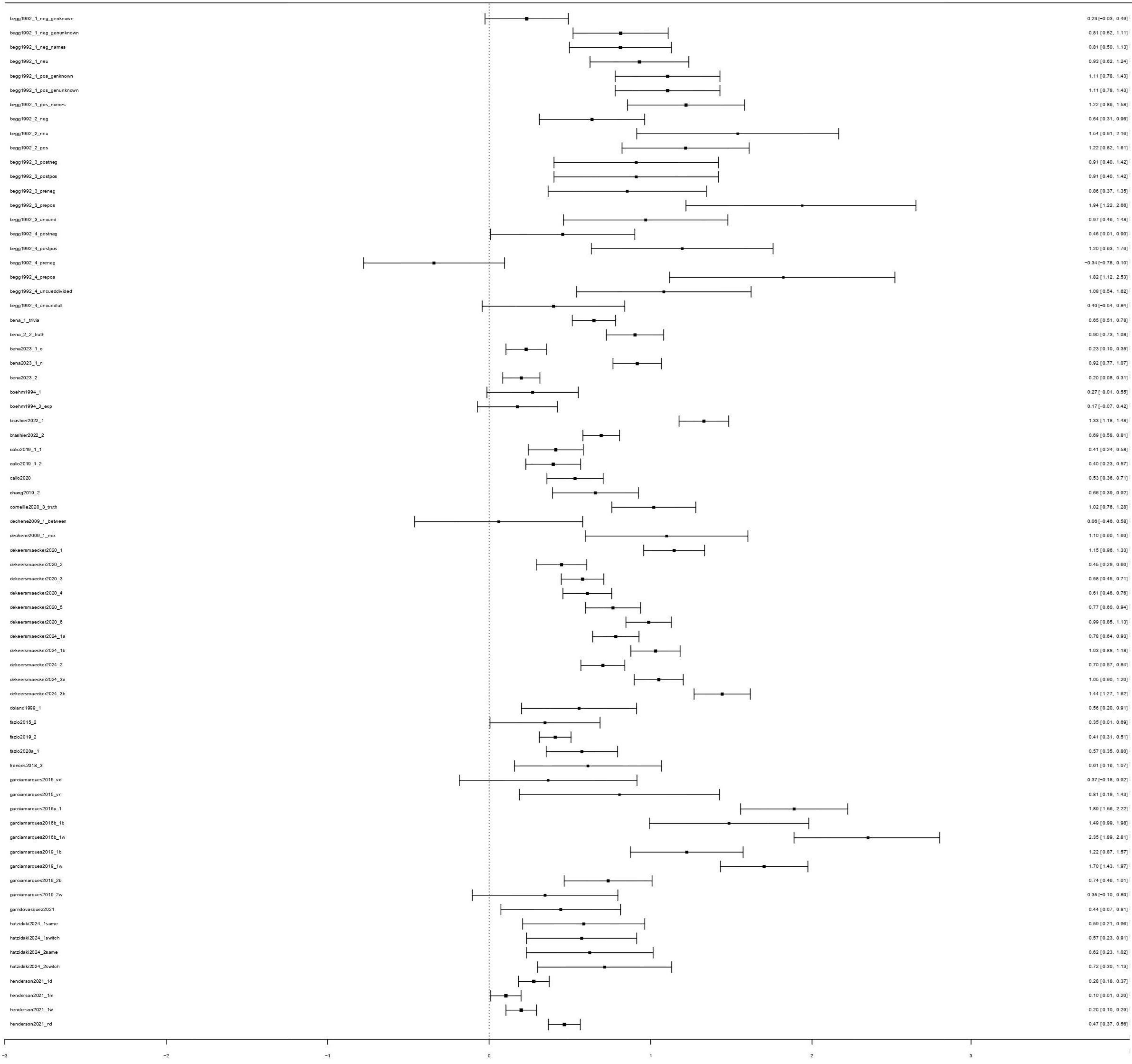

Figure S6d. Forest plot by Item type: Statements

This forest plot shows the fourth group of individual study effect sizes (Hedges' g) with corresponding 95% confidence intervals for statements-based items. Effect sizes are based on the first imputed dataset and are displayed for visual comparison across studies.

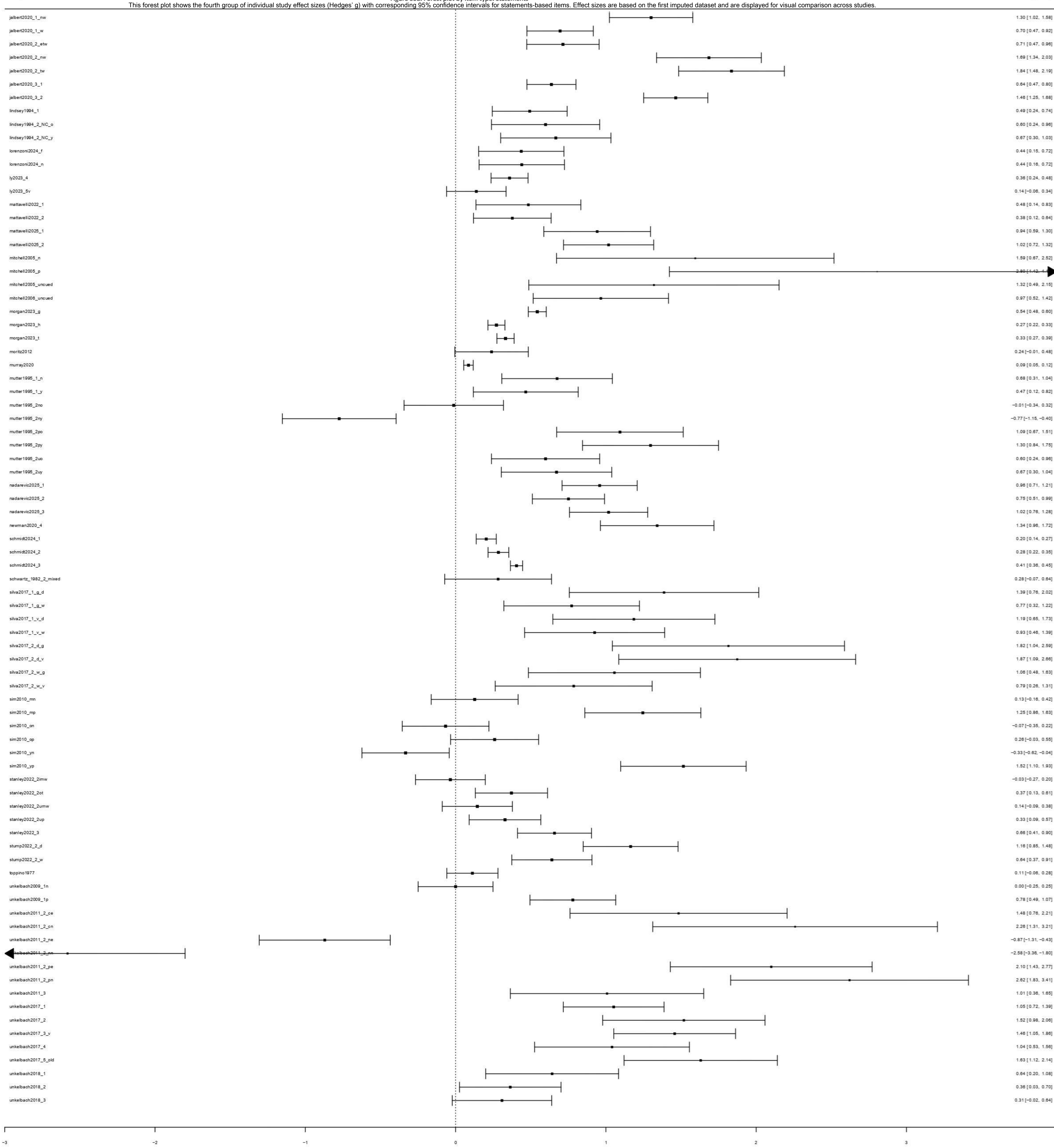

Figure S7. Forest plot by Item type: Headlines

This forest plot shows the individual study effect sizes (Hedges' g) with corresponding 95% confidence intervals for headline-based items. Effect sizes are based on the first imputed dataset and are displayed for visual comparison across studies.

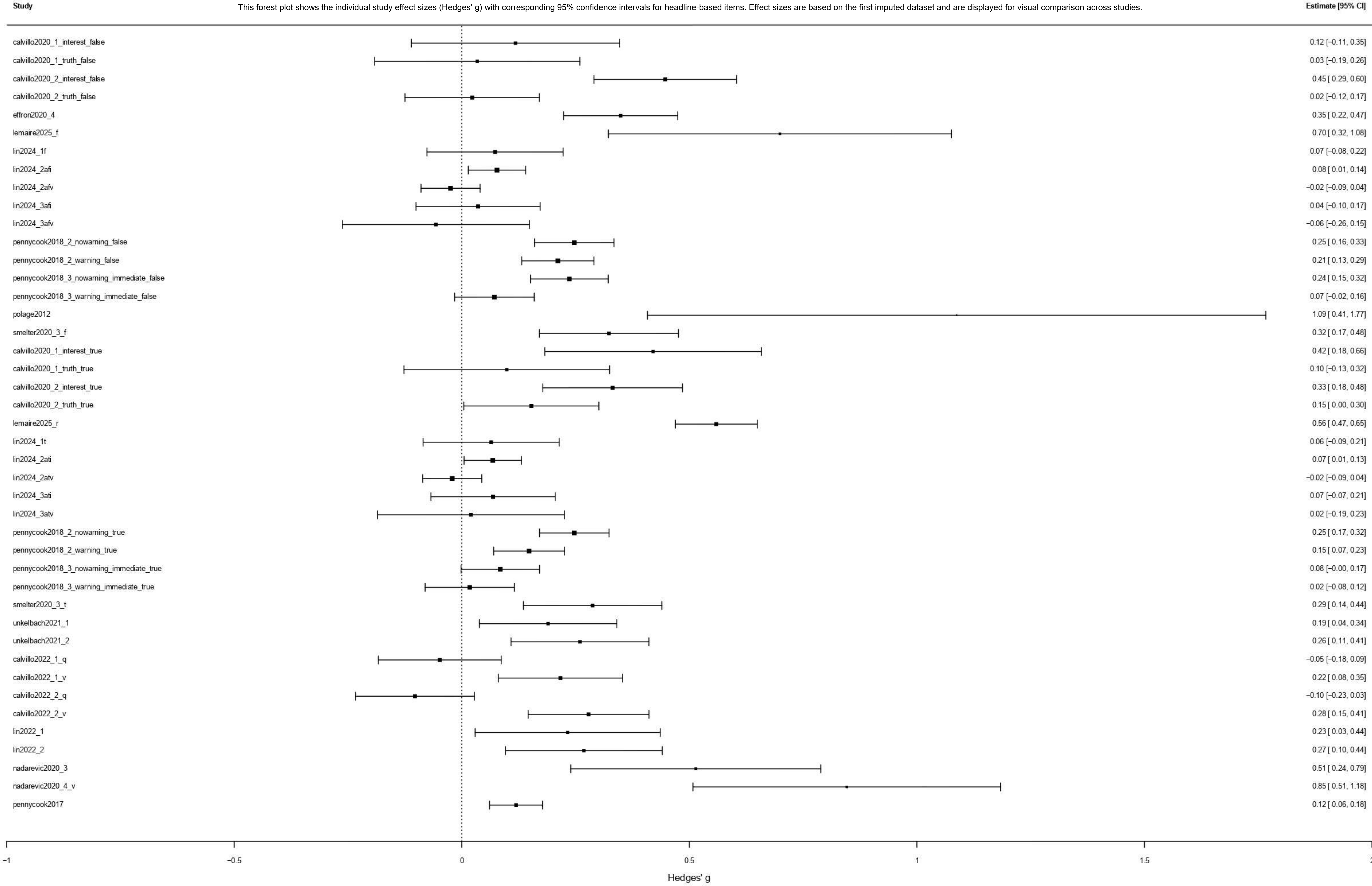

Figure S8. Forest plot by Exposure task: Veracity ratings

This forest plot shows the individual study effect sizes (Hedges' g) with corresponding 95% confidence intervals for studies using veracity ratings during the exposure phase. Effect sizes are based on the first imputed dataset and are displayed for visual comparison across studies.

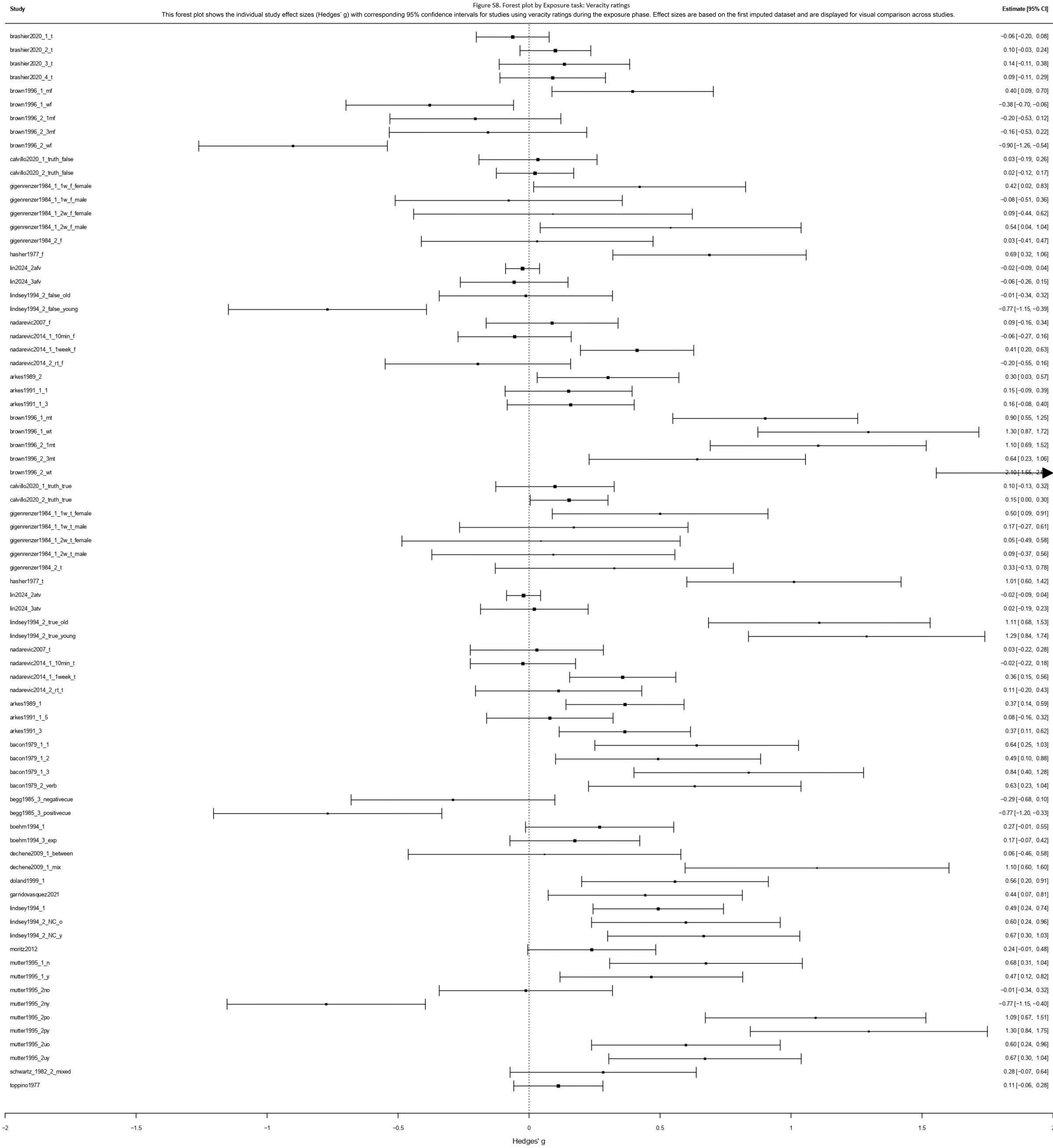

Figure S9a. Forest plot by Exposure task: Irrelevant task

This forest plot shows the first group of individual study effect sizes (Hedges' g) with corresponding 95% confidence intervals for studies using irrelevant tasks during the exposure phase. Effect sizes are based on the first imputed dataset and are displayed for visual comparison across studies.

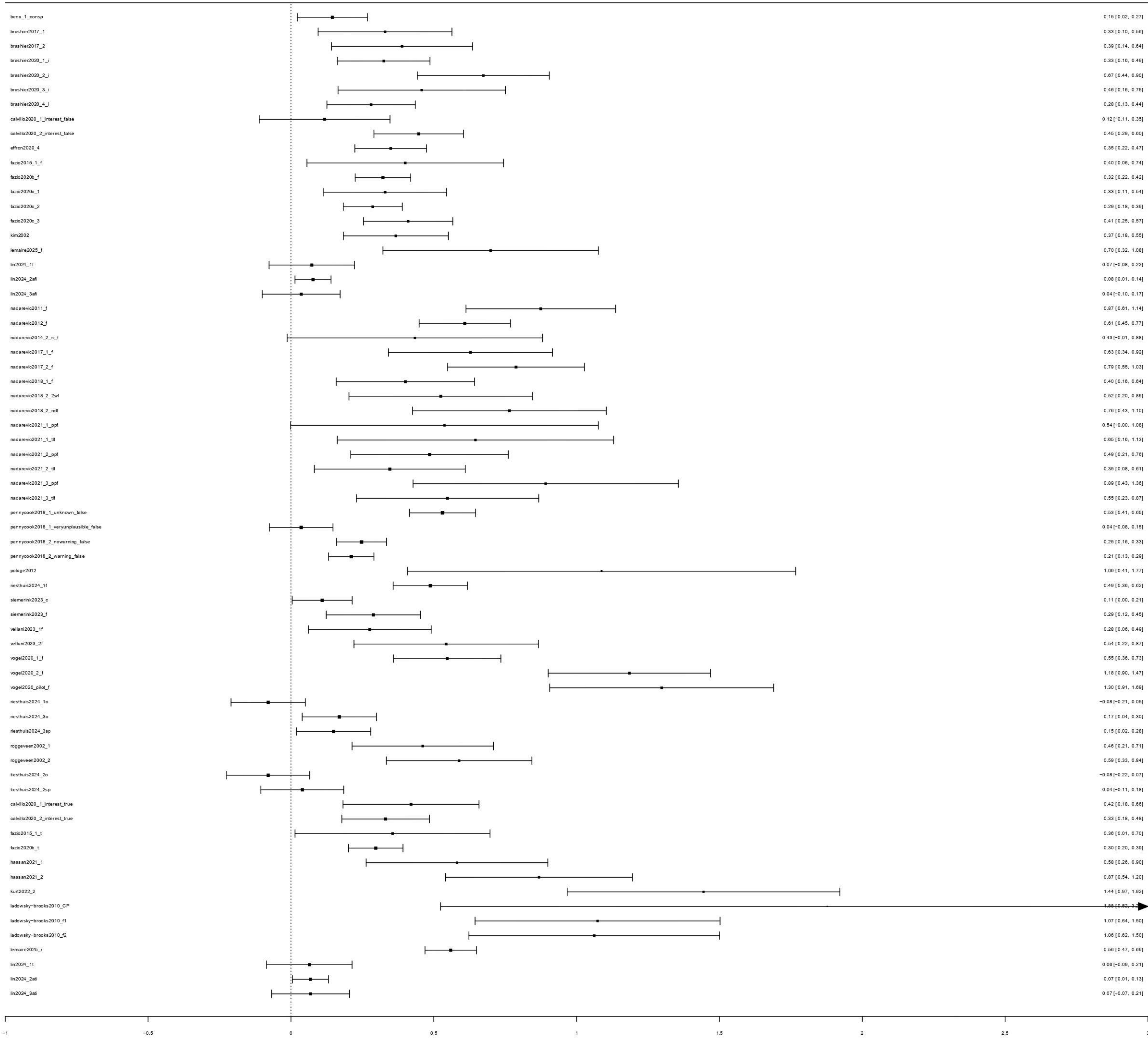

Figure S9b. Forest plot by Exposure task: Irrelevant task

This forest plot shows the second group of individual study effect sizes (Hedges' g) with corresponding 95% confidence intervals for studies using irrelevant tasks during the exposure phase. Effect sizes are based on the first imputed dataset and are displayed for visual comparison across studies.

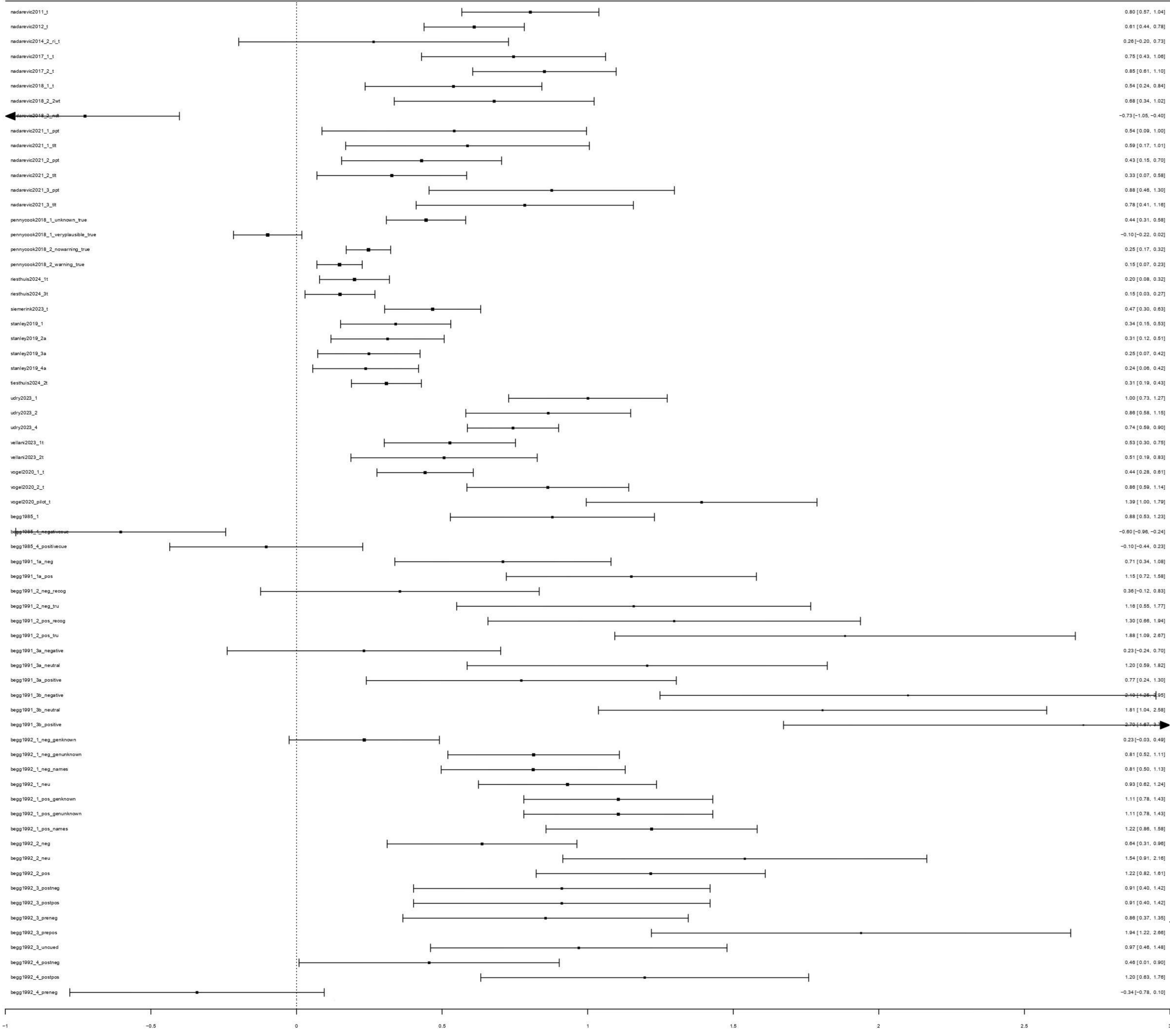

Figure S9c. Forest plot by Exposure task: Irrelevant task

This forest plot shows the third group of individual study effect sizes (Hedges' g) with corresponding 95% confidence intervals for studies using irrelevant tasks during the exposure phase. Effect sizes are based on the first imputed dataset and are displayed for visual comparison across studies.

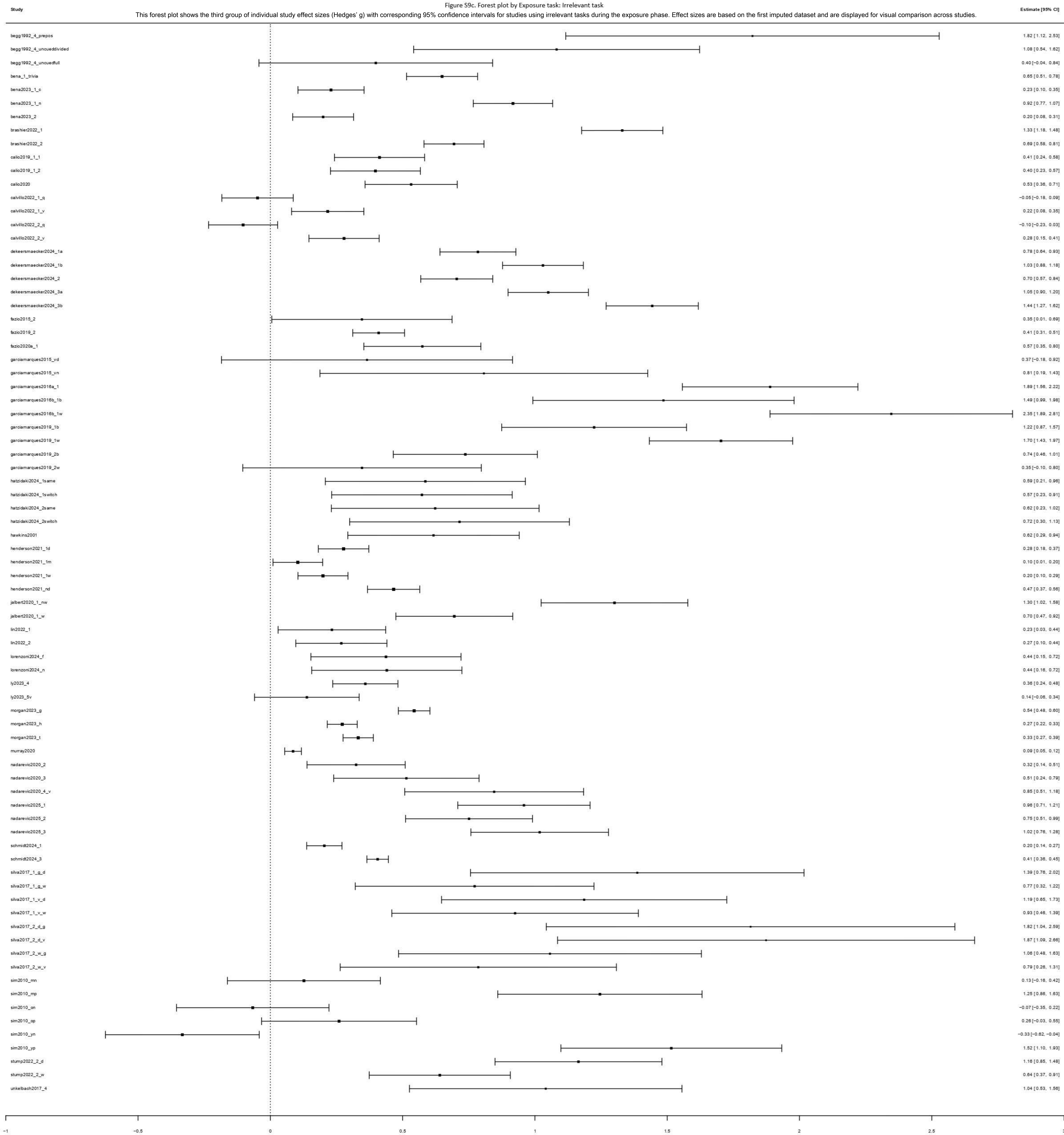

Figure S10. Forest plot by Exposure task: Passive processing

This forest plot shows the individual study effect sizes (Hedges' g) with corresponding 95% confidence intervals for studies using passive processing tasks during the exposure phase. Effect sizes are based on the first imputed dataset and are displayed for visual comparison across studies.

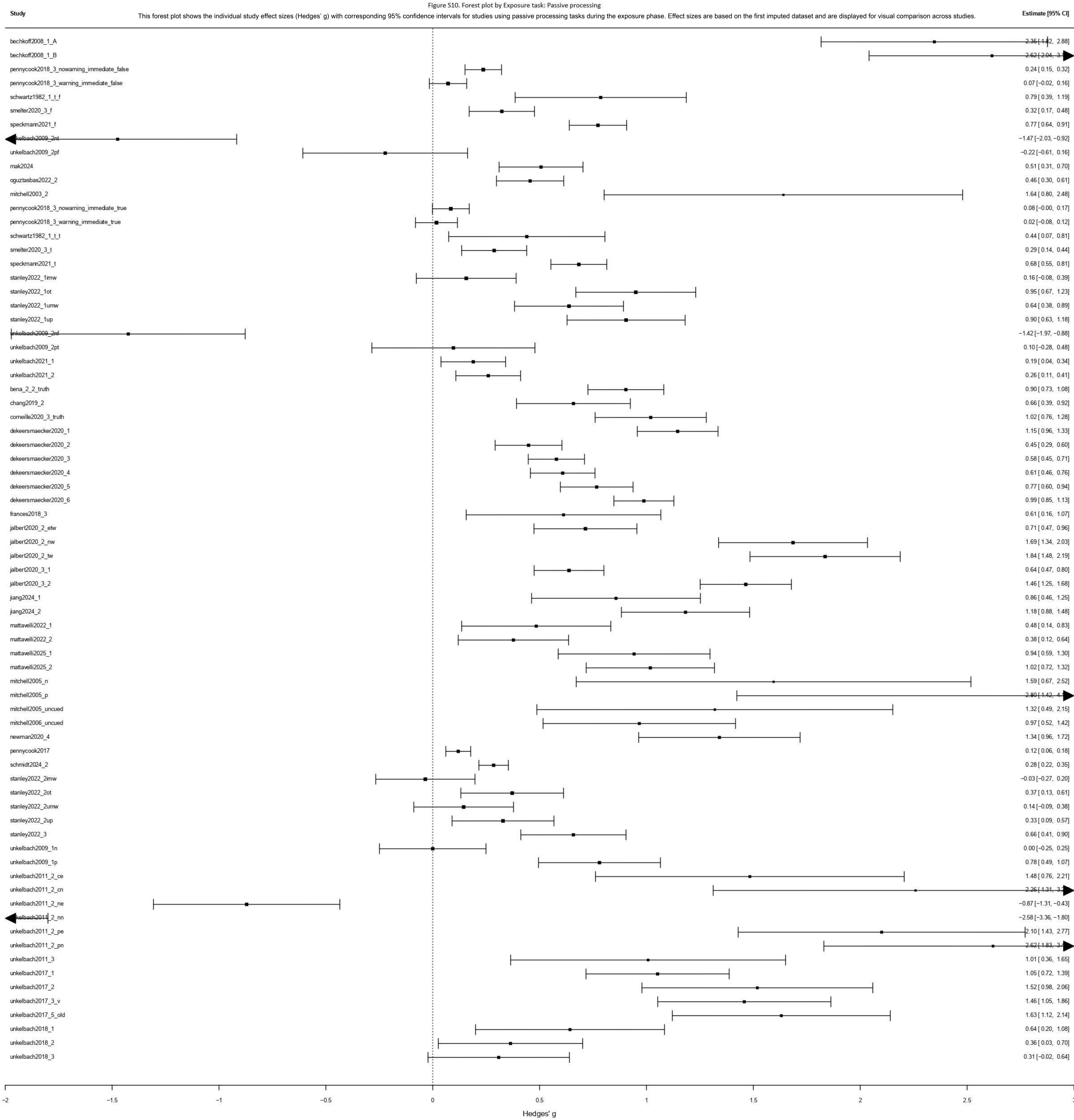

Figure S11. Forest plot by Veracity cues: True cue

This forest plot shows the individual study effect sizes (Hedges' g) with corresponding 95% confidence intervals for studies using a true veracity cue. Effect sizes are based on the first imputed dataset and are displayed for visual comparison across studies.

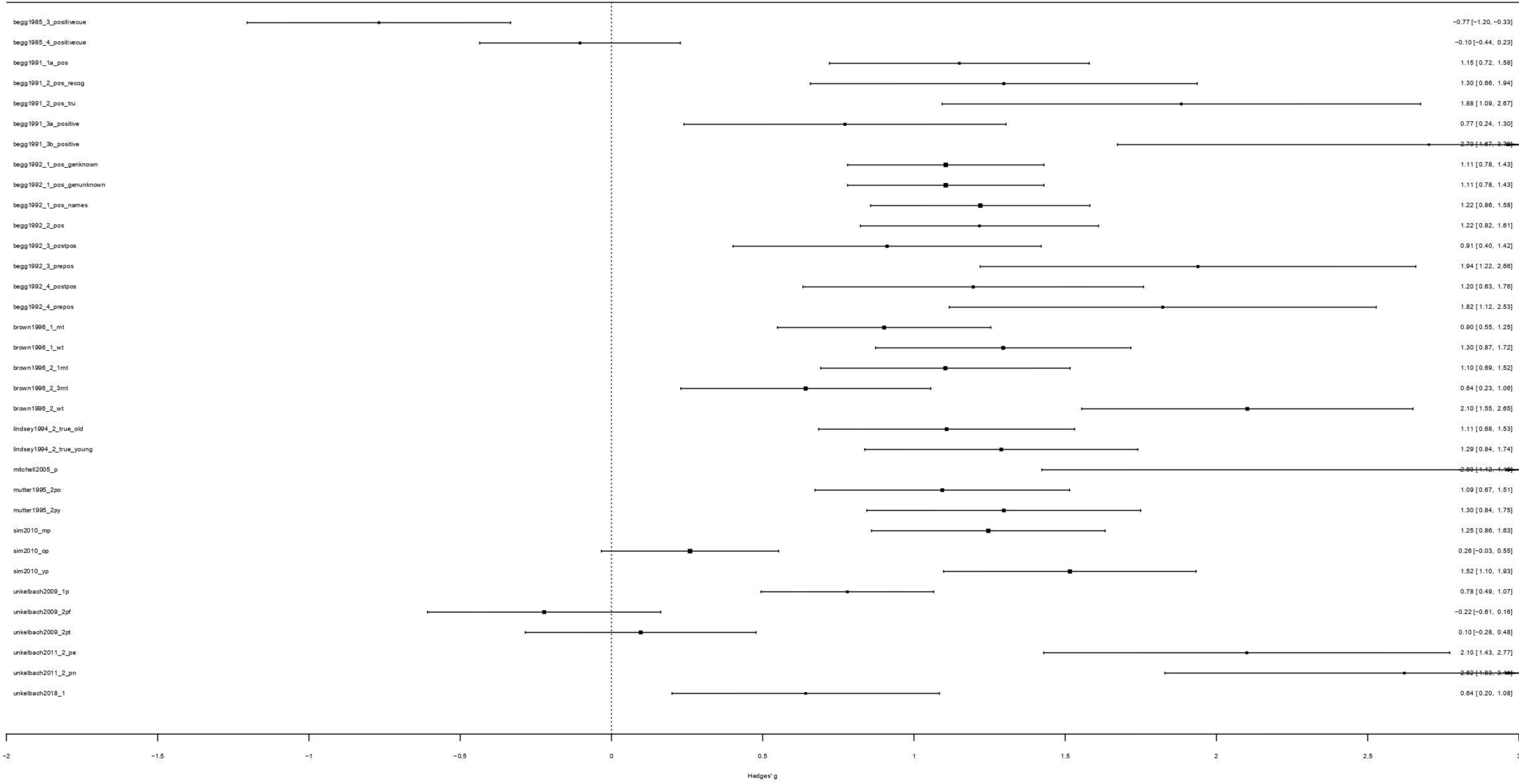

Figure S12a. Forest plot by Veracity cues: Uncued

This forest plot shows the first group of individual study effect sizes (Hedges' g) with corresponding 95% confidence intervals for studies using no veracity cue. Effect sizes are based on the first imputed dataset and are displayed for visual comparison across studies.

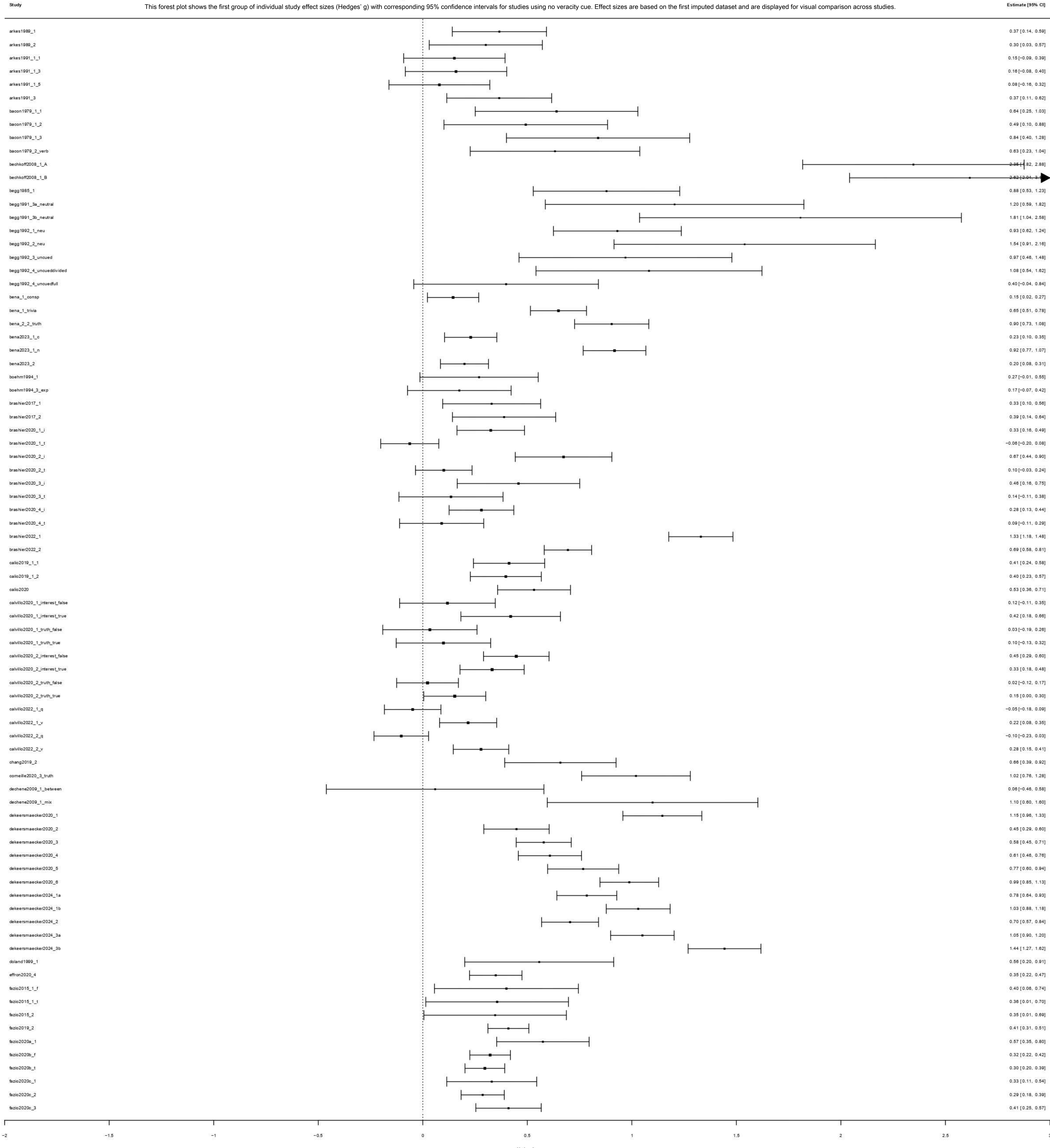

Figure S12b. Forest plot by Veracity cues: Uncued

This forest plot shows the second group of individual study effect sizes (Hedges' g) with corresponding 95% confidence intervals for studies using no veracity cue. Effect sizes are based on the first imputed dataset and are displayed for visual comparison across studies.

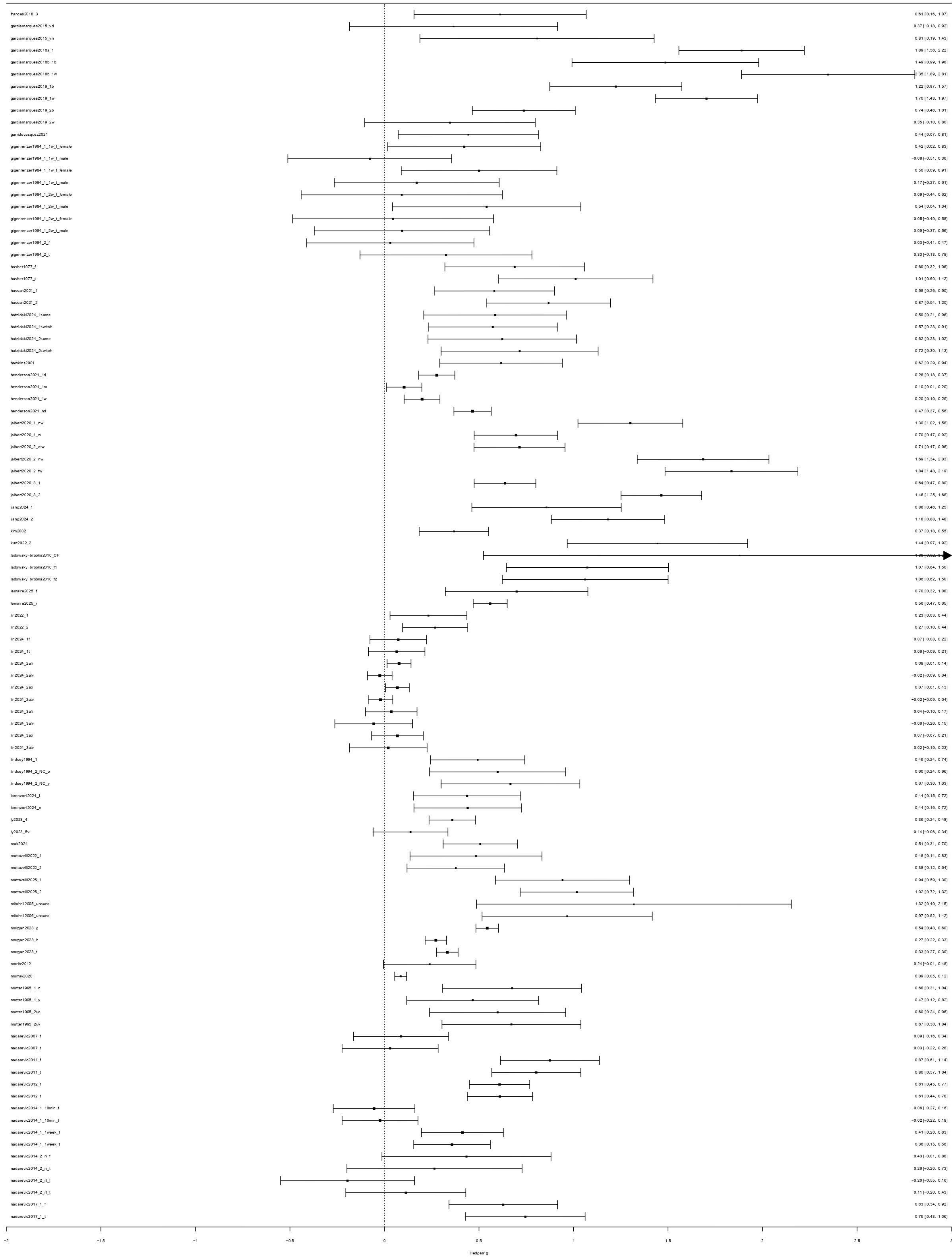

Figure S12c. Forest plot by Veracity cues: Uncued

This forest plot shows the third group of individual study effect sizes (Hedges' g) with corresponding 95% confidence intervals for studies using no veracity cue. Effect sizes are based on the first imputed dataset and are displayed for visual comparison across studies.

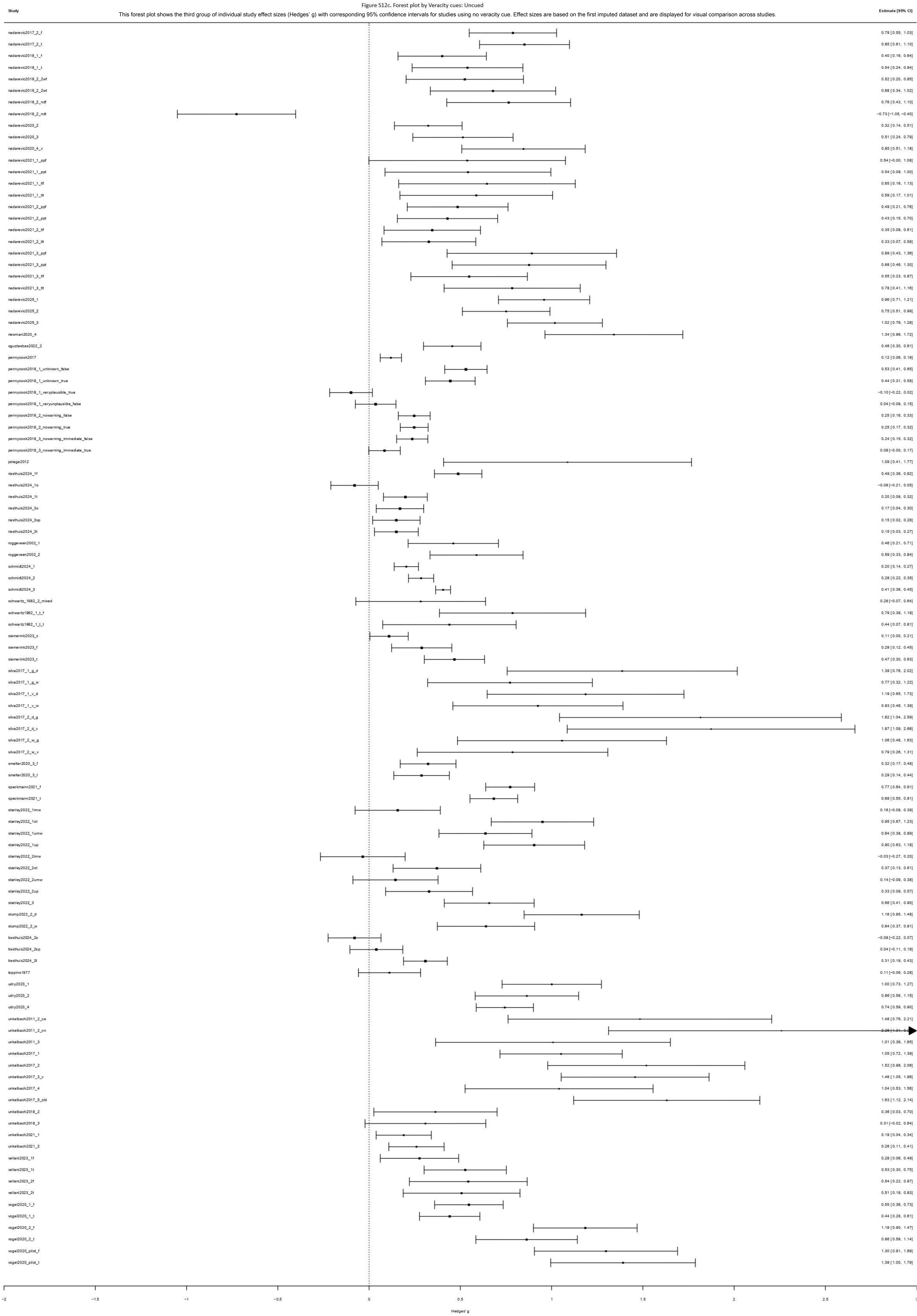

Figure S13. Forest plot by Veracity cues: False cue

This forest plot shows the individual study effect sizes (Hedges' g) with corresponding 95% confidence intervals for studies using false veracity cue. Effect sizes are based on the first imputed dataset and are displayed for visual comparison across studies.

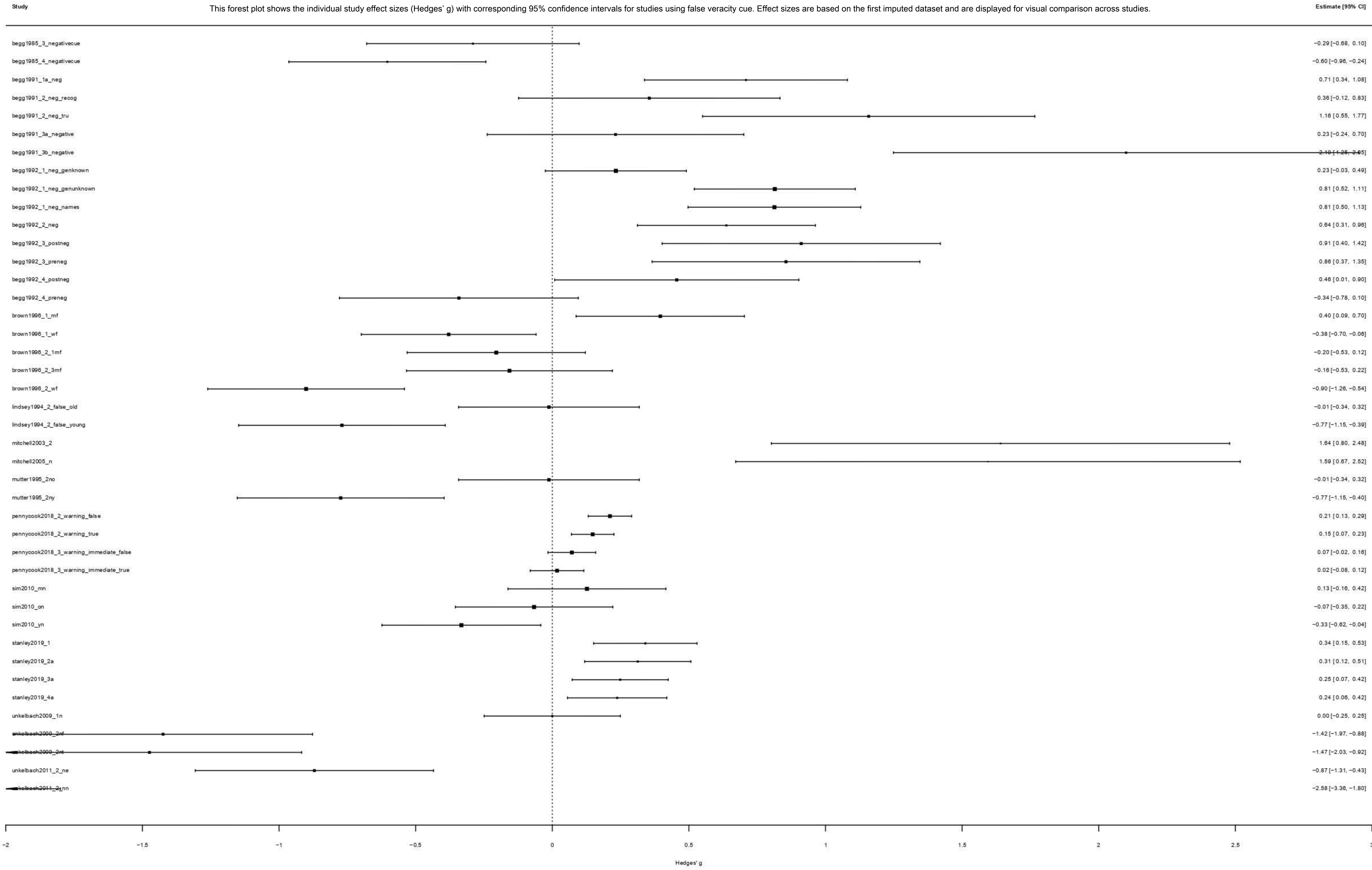

Figure S14. Forest plot by Presentation time during exposure: 5 seconds or less

This forest plot shows the individual study effect sizes (Hedges' g) with corresponding 95% confidence intervals for studies with presentation times shorter than 5 seconds during the exposure phase. Effect sizes are based on the first imputed dataset and are displayed for visual comparison across studies.

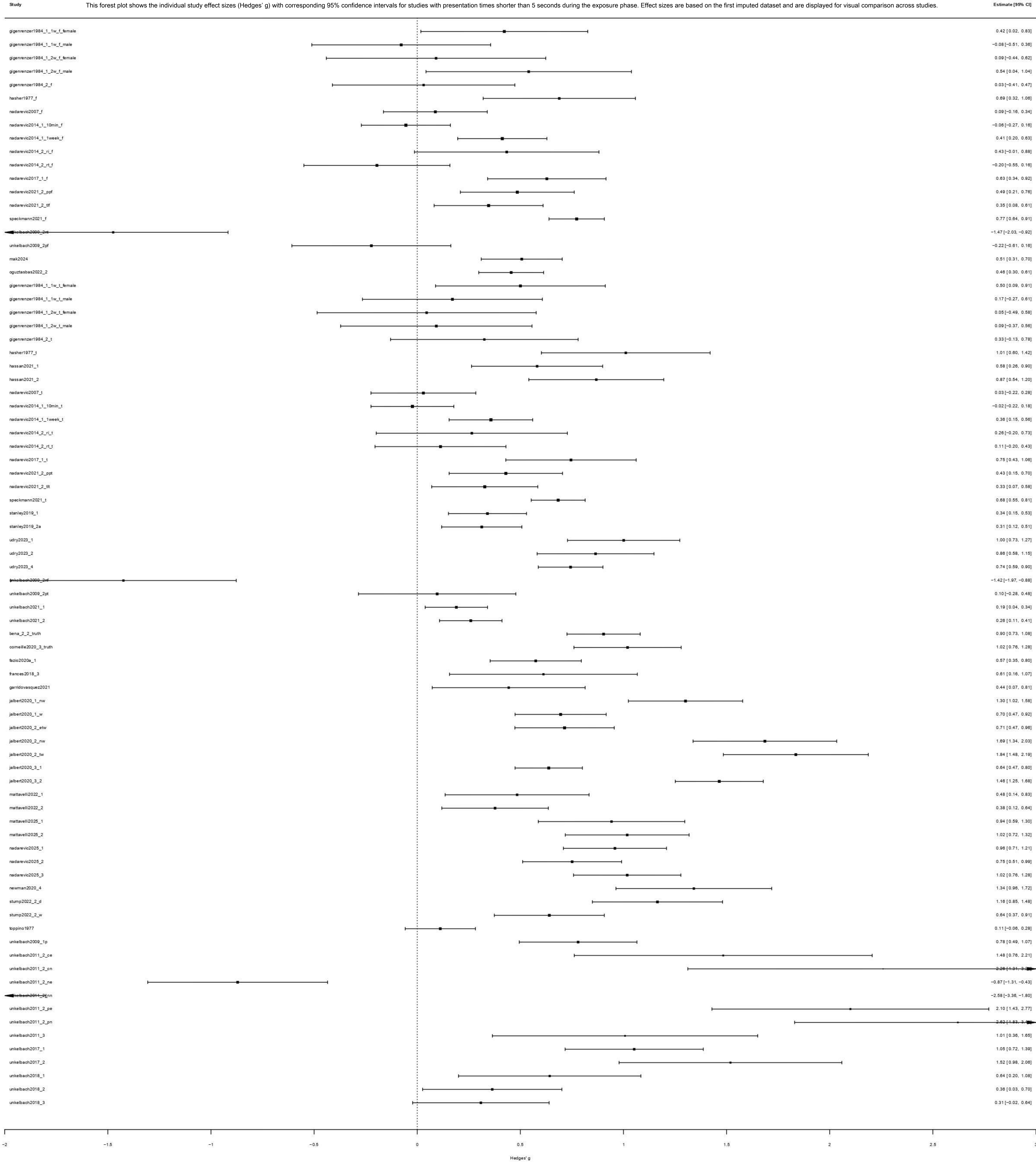

Figure S15a. Forest plot by Presentation time during exposure: Participant-paced

This forest plot shows the first group of individual study effect sizes (Hedges' g) with corresponding 95% confidence intervals for studies with participant-paced presentation times during the exposure phase. Effect sizes are based on the first imputed dataset and are displayed for visual comparison across studies.

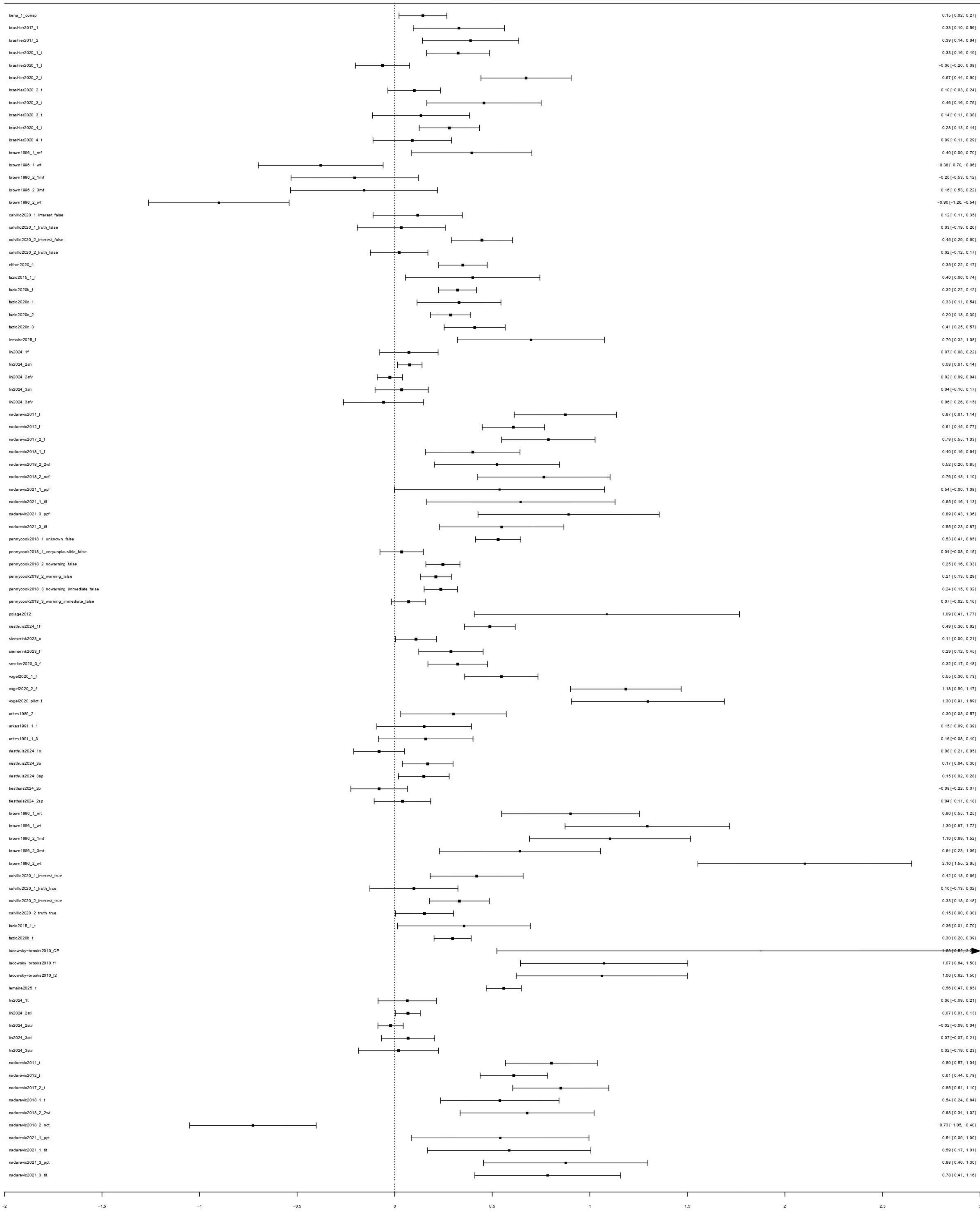

Figure S15b. Forest plot by Presentation time during exposure: Participant-paced

This forest plot shows the second group of individual study effect sizes (Hedges' g) with corresponding 95% confidence intervals for studies with participant-paced presentation times during the exposure phase. Effect sizes are based on the first imputed dataset and are displayed for visual comparison across studies.

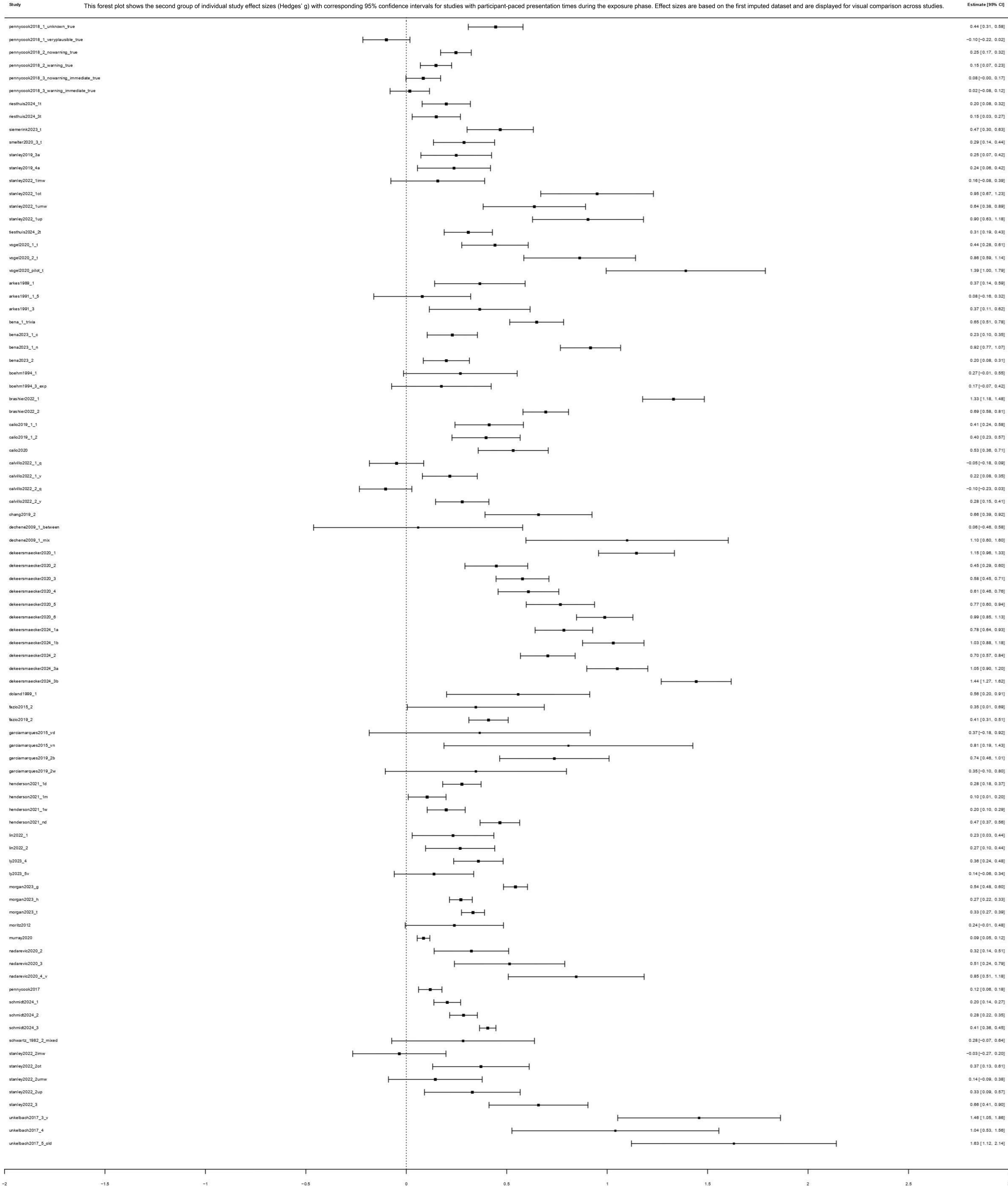

Figure S16. Forest plot by Presentation time during exposure: More than 5 seconds

This forest plot shows the individual study effect sizes (Hedges' g) with corresponding 95% confidence intervals for studies with presentation times longer than 5 seconds during the exposure phase. Effect sizes are based on the first imputed dataset and are displayed for visual comparison across studies.

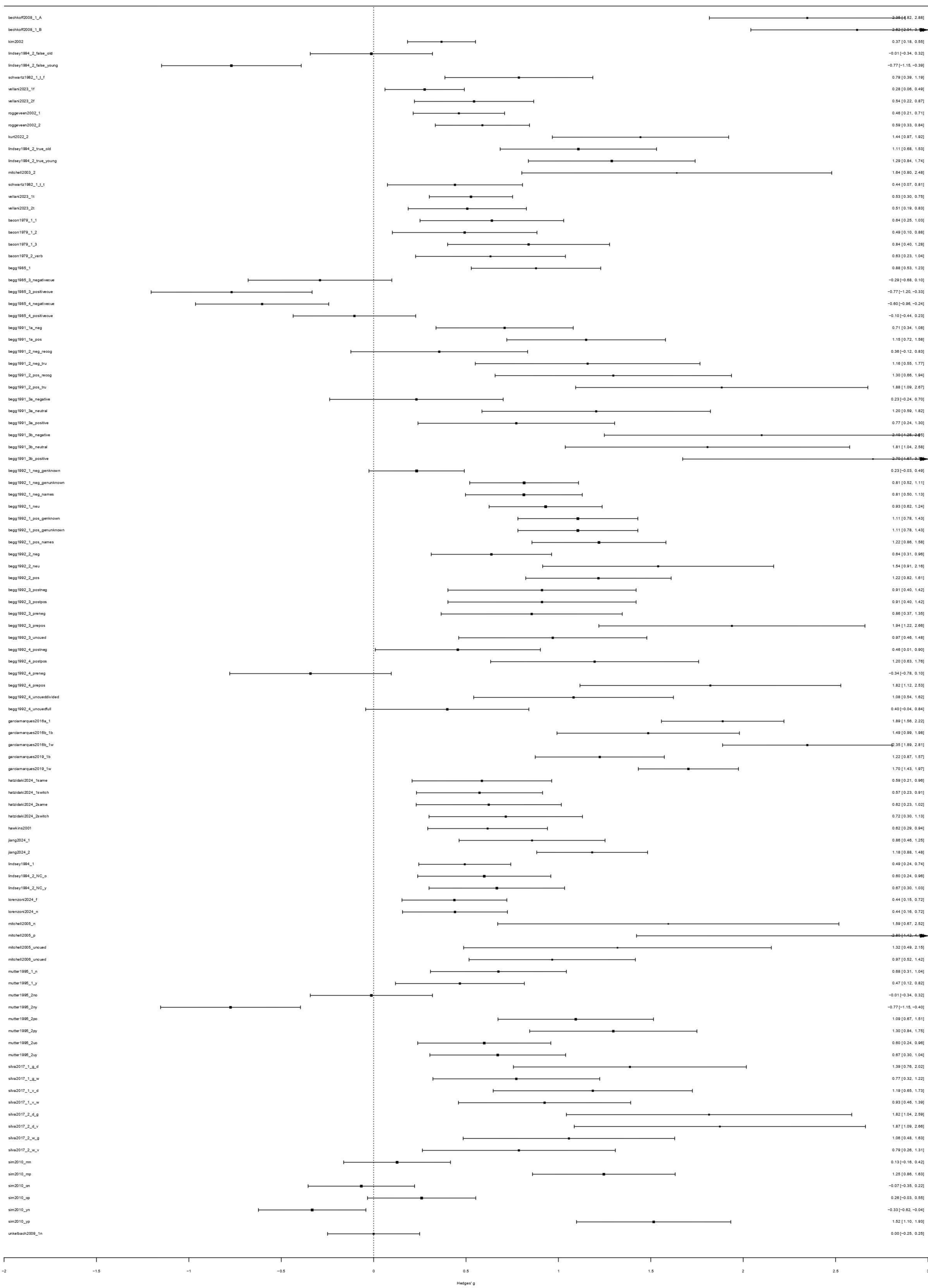

Supplementary Methods

Extensive revised keyword used in Henderson et al. (2022).

(((“illusory truth” OR “illusion* of truth” OR “induced truth effect” OR “reiteration effect” OR “tainted truth effect” OR “repetition based truth effect” OR “repetition induced increases” OR repeat OR repeated OR repeating OR repetition OR “prior exposure”) AND (true* OR truth OR “truth effect*” OR belief) AND (statement* OR items OR stimulus OR stimuli OR claim* OR judgment* OR judgement* OR rating* OR “subjective truth” OR “truth value” OR “judged validity” OR “validity ratings” OR “processing fluency” OR “fluency effect*” OR “perceptual fluency”)))

Supplementary analysis for veracity cues moderator including the type of cues.

In the main manuscript, we examined veracity cue valence (e.g., true cues, false cues, or no cue) as a moderator of the illusory truth effect. At a reviewer’s suggestion, we additionally explored whether different types of veracity cues (e.g., labels, feedback, epistemic qualifiers) differentially moderate the effect when considered jointly with valence.

Based on our re-examination of included studies, we identified five distinct cue types:

- Epistemic qualifiers (e.g., “it is certain that…,” “it is unlikely that…” preceding a statement)
- Source reliability cues (e.g., all statements voiced by a designated source are true/false)
- Labels (e.g., explicit “true”/ “false” tags)

- Immediate feedback (corrective feedback given directly after each judgment)
- Delayed feedback (corrective feedback delivered after the exposure phase or at the beginning of the test phase)

Table S3 summarizes PEESE-corrected effect sizes by cue type and valence. For comparison, the baseline (no cue) condition yielded a moderate illusory truth effect ($g = 0.43$, 95% CI [0.36, 0.50], $u = 287$).

Relative to the no-cue condition, we observed significant reductions in the illusory truth effect for false cues involving:

- Delayed feedback ($g = –1.12$, 95% CI [–1.51, –0.73], $t(292) = 5.69$, $p < .001$)
- Immediate feedback ($g = –0.91$, 95% CI [–1.44, –0.39], $t(293) = 3.31$, $p = .001$)
- Source reliability cues ($g = –0.73$, 95% CI [–0.95, –0.50], $t(319) = 6.33$, $p < .001$)
- Epistemic qualifiers ($g = –0.57$, 95% CI [–0.87, –0.29], $t(273) = 3.85$, $p < .001$)

By contrast, false labels did not reliably reduce the illusory truth effect ($b = 0.04$, 95% CI [–0.29, 0.38], $t(197) < 1$).

Direct comparisons between cue types indicated that false delayed feedback was significantly more effective than false epistemic qualifiers ($b = –0.55$, 95% CI [–1.04, –0.06], $t(263) = 2.20$, $p = .029$).

For true cues, delayed feedback increased the illusory truth effect ($b = 0.49$, 95% CI [0.09, 0.89], $t(295) = 2.42$, $p = .016$).

These exploratory analyses suggest that the type of veracity cue may interact with valence to shape the illusory truth effect. Feedback-based cues (immediate or delayed), source

reliability information, and epistemic qualifiers were generally effective in reducing the effect when signaling falsity, whereas labels were not. However, several cue type–valence combinations were represented by very few studies, limiting the reliability and generalizability of the estimates. Results should therefore be interpreted with caution.

Computation methods

We used Becker's formula as described in Morris & Deshon[2] to compute Cohen's d with repeated statements considered post-test and new statements considered pre-test as follows:

(1)

$$d = \frac{Mpost - Mpre}{SDpre}$$

Sampling variance was computed as follows:

(2)

$$v = \left[\frac{2(1-r)}{n}\right]\left(\frac{n-1}{n-3}\right)\left[1 + \frac{n}{2(1-r)}d^2\right] - \frac{d^2}{[c(n-1)]^2}$$

Small-sample correction were then applied to the estimated effect sizes of both Borenstein's and Becker's d to obtain Hedge's g. The table above describes the pooled estimates with no further correction

Supplementary References

1. Page, M. J. *et al.* The PRISMA 2020 statement: An updated guideline for reporting systematic reviews. *Int. J. Surg.* **88**, 105906 (2021).

2. Morris, S. B. & Deshon, R. P. Combining effect size estimates in meta-analysis with repeated measures and independent-groups designs. *Psychol. Methods* **7**, 105–125 (2002).

Reference for all studies included in the meta-analysis.

Arkes, H. R., Hackett, C. & Boehm, L. The generality of the relation between familiarity and judged validity. *Journal of Behavioral Decision Making* **2**, 81–94 (1989).

Arkes, H. R., Boehm, L. E. & Xu, G. Determinants of judged validity. *Journal of Experimental Social Psychology* **27**, 576–605 (1991).

Bacon, F. T. Credibility of repeated statements: Memory for trivia. *Journal of Experimental Psychology: Human Learning and Memory* **5**, 241 (1979).

Bechkoff, J. R. *Proprioception and the Truth Effect: A Case in Favor of the Cartesian Model of Information Processing*. Doctoral dissertation, University of Cincinnati (2008).

Begg, I., Armour, V. & Kerr, T. On believing what we remember. *Canadian Journal of Behavioural Science/Revue canadienne des sciences du comportement* **17**, 199 (1985).

Begg, I. & Armour, V. Repetition and the ring of truth: Biasing comments. *Canadian Journal of Behavioural Science/Revue canadienne des sciences du comportement* **23**, 195 (1991).

Begg, I. M., Anas, A. & Farinacci, S. Dissociation of processes in belief: Source recollection, statement familiarity, and the illusion of truth. *Journal of Experimental Psychology: General* **121**, 446 (1992).

Béna, J., Corneille, O., Mierop, A. & Unkelbach, C. Robustness tests provide further support for an ecological account of the truth and fake news by repetition effects. *PsyArXiv* (2021).

Béna, J., Rihet, M., Carreras, O. & Terrier, P. Repetition could increase the perceived truth of conspiracy theories. *Psychonomic Bulletin & Review* **30**, 1–10 (2023).

Béna, J., Rouard, M. & Corneille, O. You won't believe it! Truth judgments for clickbait headlines benefit (but less so) from prior exposure. *Applied Cognitive Psychology* **37**, 1418–1429 (2023).

Boehm, L. E. The validity effect: A search for mediating variables. *Personality and Social Psychology Bulletin* **20**, 285–293 (1994).

Brashier, N. M., Eliseev, E. D. & Marsh, E. J. An initial accuracy focus prevents illusory truth. *Cognition* **194**, 104054 (2020).

Brashier, N. M. & Rand, D. G. Illusory truth occurs even with incentives for accuracy. *PsyArXiv* (2021).

Brashier, N. M., Umanath, S., Cabeza, R. & Marsh, E. J. Competing cues: Older adults rely on knowledge in the face of fluency. *Psychology and Aging* **32**, 331–337 (2017).

Brown, A. S. & Nix, L. A. Turning lies into truths: Referential validation of falsehoods. *Journal of Experimental Psychology: Learning, Memory, and Cognition* **22**, 1088 (1996).

Calio, F. *Untersuchungen zur zeitlichen Stabilität und zur Vermeidbarkeit der Wahrheitsillusion*. Doctoral dissertation, Heinrich-Heine-Universität Düsseldorf (2019).

Calio, F., Nadarevic, L. & Musch, J. How explicit warnings reduce the truth effect: A multinomial modeling approach. *Acta Psychologica* **211**, 103185 (2020).

Calvillo, D. P. & Harris, J. D. Exposure to headlines as questions reduces illusory truth for subsequent headlines. *Journal of Applied Research in Memory and Cognition* **12**, 335–343 (2023).

Calvillo, D. P. & Smelter, T. J. An initial accuracy focus reduces the effect of prior exposure on perceived accuracy of news headlines. *Cognitive Research: Principles and Implications* **5**, 55 (2020).

Chang, Y. *Is the Plausibility Account of the Illusion of Truth Effect Plausible?* Doctoral dissertation, The University of North Carolina at Greensboro (2019).

Corneille, O., Mierop, A. & Unkelbach, C. Repetition increases both the perceived truth and fakeness of information: An ecological account. *Cognition* **205**, 104470 (2020).

Dechêne, A., Stahl, C., Hansen, J. & Wänke, M. Mix me a list: Context moderates the truth effect and the mere-exposure effect. *Journal of Experimental Social Psychology* **45**, 1117–1122 (2009).

De Keersmaecker, J., Dunning, D., Pennycook, G., Rand, D. G., Sanchez, C., Unkelbach, C. & Roets, A. Investigating the robustness of the illusory truth effect across individual differences in cognitive ability, need for cognitive closure, and cognitive style. *Personality and Social Psychology Bulletin* **46**, 204–215 (2020).

Unkelbach, C. & Roets, A. Truth-by-repetition across languages. *Journal of Applied Research in Memory and Cognition* **14**, 167–177 (2024).

Doland, C. A. *Repeating is believing: An investigation of the illusory truth effect*. Doctoral dissertation, State University of New York at Albany (1999).

Effron, D. A. & Raj, M. Misinformation and morality: Encountering fake-news headlines makes them seem less unethical to publish and share. *Psychological Science* **31**, 75–87 (2020).

Fazio, L. K. Repetition increases perceived truth even for known falsehoods. *Collabra: Psychology* 6, **38** (2020).

Fazio, L. K. *Preventing the Illusory Truth Effect: When Repetition Does Not Increase Perceived Truth*. Unpublished manuscript (2020).

Fazio, L. K., Brashier, N. M., Payne, B. K. & Marsh, E. J. Knowledge does not protect against illusory truth. *Journal of Experimental Psychology: General* **144**, 993–1002 (2015).

Fazio, L. K., Rand, D. G. & Pennycook, G. Repetition increases perceived truth equally for plausible and implausible statements. *Psychonomic Bulletin & Review* **26**, 1705–1710 (2019).

Fazio, L. K. & Sherry, C. L. The effect of repetition on truth judgments across development. *Psychological Science* **31**, 11 (2020).

Frances, C., Costa, A. & Baus, C. On the effects of regional accents on memory and credibility. *Acta Psychologica* **186**, 63–70 (2018).

Garcia-Marques, T., Prada, M. & Mackie, D. M. Familiarity increases subjective positive affect even in non-affective and non-evaluative contexts. *Motivation and Emotion* **40**, 638–645 (2016).

Garcia-Marques, T., Silva, R. R. & Mello, J. Judging the truth-value of a statement in and out of a deep processing context. *Social Cognition* **34**, 40–54 (2016).

Garcia-Marques, T., Silva, R. R., Mello, J. & Hansen, J. Relative to what? Dynamic updating of fluency standards and between-participants illusions of truth. *Acta Psychologica* **195**, 71–79 (2019).

Garcia-Marques, T., Silva, R. R., Reber, R. & Unkelbach, C. Hearing a statement now and believing the opposite later. *Journal of Experimental Social Psychology* **56**, 126–129 (2015).

Garrido-Vásquez, P. & Rock, T. Judgments of truth are independently modulated by affect and repetition. *PsyArXiv* (2021).

Gigerenzer, G. External validity of laboratory experiments: The frequency-validity relationship. *The American Journal of Psychology* **97**, 185–195 (1984).

Hasher, L., Goldstein, D. & Toppino, T. Frequency and the conference of referential validity. *Journal of Verbal Learning and Verbal Behavior* **16**, 107–112 (1977).

Hassan, A. & Barber, S. J. The effects of repetition frequency on the illusory truth effect. *Cognitive Research: Principles and Implications* **6**, 1–12 (2021).

Hatzidaki, A., Santesteban, M. & Navarrete, E. Illusory truth effect across languages and scripts. *Psychonomic Bulletin & Review* **31**, 1–9 (2024).

Hawkins, S. A., Hoch, S. J. & Meyers-Levy, J. Low-involvement learning: Repetition and coherence in familiarity and belief. *Journal of Consumer Psychology* **11**, 1–11 (2001).

Henderson, E. L., Simons, D. J. & Barr, D. J. The trajectory of truth: A longitudinal study of the illusory truth effect. *Journal of Cognition* **4**, 29 (2021).

Jalbert, M., Schwarz, N. & Newman, E. Only half of what I'll tell you is true: Expecting to encounter falsehoods reduces illusory truth. *Journal of Applied Research in Memory and Cognition* **9**, 602–613 (2020).

Jiang, Y., Schwarz, N., Reynolds, K. J. & Newman, E. J. Repetition increases belief in climate-skeptical claims, even for climate science endorsers. *PLOS ONE* **19**, e0307294 (2024).

Kim, C. *The role of individual differences in general skepticism in the illusory truth effect*. Doctoral dissertation, University of Cincinnati (2002).

Kurt, E. H. *The Effect of Repeated Exposure, Picture Presence and Context Reinstatement on Truth Judgments*. Master's thesis, Bilkent University (2022).

Ladowsky-Brooks, R. L. The truth effect in relation to neuropsychological functioning in traumatic brain injury. *Brain Injury* **24**, 1343–1349 (2010).

Lemaire, M., Ye, S., Le Stanc, L., Borst, G. & Cassotti, M. The development of media truth discernment and fake news detection is related to the development of reasoning during adolescence. *Scientific Reports* **15**, 6854 (2025).

Lin, J. *Medium of mass misinformation: Repetition increases people's rating of truth for real and satirical headlines*. Doctoral dissertation, The University of Waikato (2022).

Lin, H., Savio, M. T., Huang, X., Steiger, M., Guevara, R. L., Szostak, D. *et al.* Accuracy prompts protect professional content moderators from the illusory truth effect. *PNAS Nexus* **3**, pgae481 (2024).

Lindsey, S. *Aging and the Truth Effect in Validity Judgment*. Master's dissertation, Western Kentucky University (1994).

Lorenzoni, A., Faccio, R. & Navarrete, E. Does foreign-accented speech affect credibility? Evidence from the illusory-truth paradigm. *Journal of Cognition* **7**, 26 (2024).

Ly, D. P., Newman, E. J. & Bernstein, D. M. Repetition effects in memory and truth assessments through the lens of transfer-appropriate processing. *Memory* **31**, 328–345 (2023).

Mattavelli, S., Corneille, O. & Unkelbach, C. Truth by repetition … without repetition: Testing the effect of instructed repetition on truth judgments. *Journal of Experimental Psychology: Learning, Memory, and Cognition* **49**, 1264–1279 (2023).

Mak, C. W. & Chooi, W. T. Fluency trumps working memory capacity in the truth effect: Testing the relationship between the repetition-based truth effect and working memory capacity (WMC) online among multilingual young adults in Malaysia. *Journal of Cognitive Psychology* **36**, 220–233 (2024).

Mattavelli, S., Brambilla, M. & Unkelbach, C. *Repeating Statements Increases Source Credibility*. Unpublished manuscript (2025).

Mitchell, J. P. *Asymmetries in the processing of true and false information*. Doctoral dissertation, Harvard University (2003).

Mitchell, J. P., Dodson, C. S. & Schacter, D. L. fMRI evidence for the role of recollection in suppressing misattribution errors: The illusory truth effect. *Journal of Cognitive Neuroscience* **17**, 800–810 (2005).

Mitchell, J. P., Sullivan, A. L., Schacter, D. L. & Budson, A. E. Misattribution errors in Alzheimer's disease: The illusory truth effect. *Neuropsychology* **20**, 185 (2006).

Morgan, J. C. & Cappella, J. N. The effect of repetition on the perceived truth of tobacco-related health misinformation among US adults. *Journal of Health Communication* **28**, 182–189 (2023).

Moritz, S., Köther, U., Woodward, T. S., Veckenstedt, R., Dechêne, A. & Stahl, C. Repetition is good? An Internet trial on the illusory truth effect in schizophrenia and nonclinical participants. *Journal of Behavior Therapy and Experimental Psychiatry* **43**, 1058–1063 (2012).

Murray, S., Stanley, M., McPhetres, J., Pennycook, G. & Seli, P. "I've said it before and I will say it again": Repeating statements made by Donald Trump increases perceived truthfulness for individuals across the political spectrum. *PsyArXiv* (2020).

Mutter, S. A., Lindsey, S. E. & Pliske, R. M. Aging and credibility judgment. *Aging, Neuropsychology, and Cognition* **2**, 89–107 (1995).

Nadarevic, L. *A failed replication of the truth effect*. OSF (2007).

Nadarevic, L. & Aßfalg, A. Unveiling the truth: Warnings reduce the repetition-based truth effect. *Psychological Research* **81**, 814–826 (2017).

Nadarevic, L. & Erdfelder, E. Initial judgment task and delay of the final validity-rating task moderate the truth effect. *Consciousness and Cognition* **23**, 74–84 (2014).

Nadarevic, L. & Erdfelder, E. On the relationship between recognition judgments and truth judgments: Memory states moderate the recognition-based truth effect. *Journal of Experimental Psychology: Learning, Memory, and Cognition* (2025).

Nadarevic, L., Meckler, M. & Schmidt, A. Are there interindividual differences of the truth effect? An investigation of different personality. OSF (2012).

Nadarevic, L., Plier, S., Thielmann, I. & Darancó, S. Foreign language reduces the longevity of the repetition-based truth effect. *Acta Psychologica* **191**, 149–159 (2018).

Nadarevic, L., Reber, R., Helmecke, A. J. & Köse, D. Perceived truth of statements and simulated social media postings: An experimental investigation of source credibility, repeated exposure, and presentation format. *Cognitive Research: Principles and Implications* **5**, 1–16 (2020).

Nadarevic, L. & Rinnewitz, L. Decision mode instructions do not moderate the truth effect. OSF (2011).

Nadarevic, L., Schnuerch, M. & Stegemann, M. J. Judging fast and slow: The truth effect does not increase under time-pressure conditions. *Judgment and Decision Making* **16** (2021).

Newman, E. J., Jalbert, M. C., Schwarz, N. & Ly, D. P. Truthiness, the illusory truth effect, and the role of need for cognition. *Consciousness and Cognition* **78**, 102866 (2020).

Oğuz Taşbaş, E. H. & Unkelbach, C. Repeating stereotypes: Increased belief and subsequent discrimination. *European Journal of Social Psychology* **52**, 528–537 (2022).

Pennycook, G., Cannon, T. D. & Rand, D. G. Prior exposure increases perceived accuracy of fake news. *Journal of Experimental Psychology: General* **147**, 1865–1880 (2018).

Pennycook, G. & Rand, D. G. *The illusory truth effect for fake news is similar regardless of format*. Unpublished data (2017).

Polage, D. C. Making up history: False memories of fake news stories. *Europe's Journal of Psychology* **8**, 245–250 (2012).

Riesthuis, P. & Woods, J. "That's just like, your opinion, man": The illusory truth effect on opinions. *Psychological Research* **88**, 284–306 (2024).

Roggeveen, A. L. & Johar, G. V. Perceived source variability versus familiarity: Testing competing explanations for the truth effect. *Journal of Consumer Psychology* **12**, 81–91 (2002).

Schmidt, O. & Heck, D. W. The relevance of syntactic complexity for truth judgments: A registered report. *Consciousness and Cognition* **117**, 103623 (2024).

Schwartz, M. Repetition and rated truth value of statements. *The American Journal of Psychology* **95**, 393–407 (1982).

Siemerink, M. *Repetition As a Weapon: What Is The Effect of the Illusory Truth Effect on Conspiracy Theories and Their Debunking?* Unpublished Master's thesis (2023).

Silva, R. R., Garcia-Marques, T. & Reber, R. The informative value of type of repetition: Perceptual and conceptual fluency influences on judgments of truth. *Consciousness and Cognition* **51**, 53–67 (2017).

Sim, R. *Memory mistakes and ageing: how susceptibility to false recognition and the illusory truth effect changes across the lifespan*. Unpublished Master's thesis, Massey University (2010).

Smelter, T. J. & Calvillo, D. P. Pictures and repeated exposure increase perceived accuracy of news headlines. *Applied Cognitive Psychology* **34**, 1061–1071 (2020).

Speckmann, F. & Unkelbach, C. Monetary incentives do not reduce the repetition-induced truth effect. *Psychonomic Bulletin & Review* **29**, 1–8 (2022).

Stanley, M. L., Whitehead, P. S., Marsh, E. J. & Seli, P. Prior exposure increases judged truth even during periods of mind wandering. *Psychonomic Bulletin & Review* **29**, 1997–2007 (2022).

Stanley, M. L., Yang, B. W. & Marsh, E. J. When the unlikely becomes likely: Qualifying language does not influence later truth judgments. *Journal of Applied Research in Memory and Cognition* **8**, 118–129 (2019).

Stump, A., Rummel, J. & Voss, A. Is it all about the feeling? Affective and (meta-)cognitive mechanisms underlying the truth effect. *Psychological Research* **86**, 12–36 (2022).

Toppino, T., Robertshaw, W., Hasher, L. & Goldstein, A. Frequency of occurrence and judgements of truth and falsety. Unpublished conference paper (1977).

Udry, J. & Barber, S. J. The illusory truth effect requires semantic coherence across repetitions. *Cognition* **241**, 105607 (2023).

Unkelbach, C., Bayer, M., Alves, H., Koch, A. & Stahl, C. Fluency and positivity as possible causes of the truth effect. *Consciousness and Cognition* **20**, 594–602 (2011).

Unkelbach, C. & Greifeneder, R. Experiential fluency and declarative advice jointly inform judgments of truth. *Journal of Experimental Social Psychology* **79**, 78–86 (2018).

Unkelbach, C. & Rom, S. C. A referential theory of the repetition-induced truth effect. *Cognition* **160**, 110–126 (2017).

Unkelbach, C. & Speckmann, F. Mere repetition increases belief in factually true COVID-19-related information. *Journal of Applied Research in Memory and Cognition* **10**, 241–247 (2021).

Unkelbach, C. & Stahl, C. A multinomial modeling approach to dissociate different components of the truth effect. *Consciousness and Cognition* **18**, 22–38 (2009).

Vellani, V., Zheng, S., Ercelik, D. & Sharot, T. The illusory truth effect leads to the spread of misinformation. *Cognition* **236**, 105421 (2023).

Vogel, T., Silva, R. R., Thomas, A. & Wänke, M. Truth is in the mind, but beauty is in the eye: Fluency effects are moderated by a match between fluency source and judgment dimension. *Journal of Experimental Psychology: General* **149**, 1587–1596 (2020).
