# Supplementary material for: Systematic review and meta-analysis of the evidence for an illusory truth effect and its determinants
Source: Nat Commun. 2026 Feb 27;17:3270. doi: 10.1038/s41467-026-70041-x (PMC13066098; doi:10.1038/s41467-026-70041-x)
Supplement: Supplementary file 2 — Description of Additional Supplementary Files [file 41467_2026_70041_MOESM2_ESM.pdf]

Description of Additional Supplementary Files

File Name: Supplementary Data 1
Description: Spreadsheet containing the extracted effect sizes, sample characteristics, and study-level metadata for all studies included in the meta-analysis.

File Name: Supplementary Data 2
Description: Spreadsheet containing the Risk of Bias (RoB 2) coding for all included studies.

File Name: Supplementary Data 3
Description: Spreadsheet containing sensitivity analyses of the three-level meta-regression model using minimum and maximum estimations of missing outcomes.

File Name: Supplementary Data 4
Description: Spreadsheet containing the full results of the meta-regression analyses, including contrasts with Dechêne et al. (2010).

File Name: Supplementary Data 5
Description: Spreadsheet listing all excluded studies and excluded effect sizes, with detailed justifications for exclusion.
