# Supplementary material for: Systematic review and meta-analysis of the evidence for an illusory truth effect and its determinants
Source: Nat Commun. 2026 Feb 27;17:3270. doi: 10.1038/s41467-026-70041-x (PMC13066098; doi:10.1038/s41467-026-70041-x)
Supplement: Supplementary file 8 — Reporting Summary [file 41467_2026_70041_MOESM8_ESM.pdf]

nature portfolio

Corresponding author(s): Grégoire Borst

Last updated by author(s): Feb 2, 2026

Reporting Summary

Nature Portfolio wishes to improve the reproducibility of the work that we publish. This form provides structure for consistency and transparency in reporting. For further information on Nature Portfolio policies, see our Editorial Policies and the Editorial Policy Checklist.

Statistics

For all statistical analyses, confirm that the following items are present in the figure legend, table legend, main text, or Methods section.

n/a	Confirmed	
☐	☒	The exact sample size (*n*) for each experimental group/condition, given as a discrete number and unit of measurement
☐	☒	A statement on whether measurements were taken from distinct samples or whether the same sample was measured repeatedly
☐	☒	The statistical test(s) used AND whether they are one- or two-sided<br>*Only common tests should be described solely by name; describe more complex techniques in the Methods section.*
☐	☒	A description of all covariates tested
☐	☒	A description of any assumptions or corrections, such as tests of normality and adjustment for multiple comparisons
☐	☒	A full description of the statistical parameters including central tendency (e.g. means) or other basic estimates (e.g. regression coefficient) AND variation (e.g. standard deviation) or associated estimates of uncertainty (e.g. confidence intervals)
☐	☒	For null hypothesis testing, the test statistic (e.g. *F*, *t*, *r*) with confidence intervals, effect sizes, degrees of freedom and *P* value noted<br>*Give P values as exact values whenever suitable.*
☒	☐	For Bayesian analysis, information on the choice of priors and Markov chain Monte Carlo settings
☐	☒	For hierarchical and complex designs, identification of the appropriate level for tests and full reporting of outcomes
☐	☒	Estimates of effect sizes (e.g. Cohen's *d*, Pearson's *r*), indicating how they were calculated

Our web collection on statistics for biologists contains articles on many of the points above.

Software and code

Policy information about availability of computer code

Data collection	Excel (v.2511), Zotero (v.7.0.30), Publish or Perish (v.8.2.3944)
Data analysis	Data analysis and visualization were conducted in R (v.4.4.2) running under RStudio (2024.12.1+563), with packages metafor (v.4.8.0), ggplot2 (v.3.5.1), tidyverse (v.2.0.0), ggplot2 (v.3.5.1), VIM (v.6.2.2), robvis (v.0.3.0), and mice (v.3.17.0).

For manuscripts utilizing custom algorithms or software that are central to the research but not yet described in published literature, software must be made available to editors and reviewers. We strongly encourage code deposition in a community repository (e.g. GitHub). See the Nature Portfolio guidelines for submitting code & software for further information.

Data

Policy information about availability of data

All manuscripts must include a data availability statement. This statement should provide the following information, where applicable:

- Accession codes, unique identifiers, or web links for publicly available datasets
- A description of any restrictions on data availability
- For clinical datasets or third party data, please ensure that the statement adheres to our policy

The data generated in this study is available in the Open Science Framework (OSF) repository under accession DOI: https://doi.org/10.17605/OSF.IO/2DB8S. No raw individual-level participant data were generated in this study. The processed data underlying all analyses, including extracted effect sizes, moderator codings, and imputed datasets, are available in the OSF repository. No data are subject to ethical, legal, or commercial restrictions.

Research involving human participants, their data, or biological material

Policy information about studies with human participants or human data. See also policy information about sex, gender (identity/presentation), and sexual orientation and race, ethnicity and racism.

Reporting on sex and gender	This study is a meta-analysis of previously published experimental studies and did not involve new data collection or interactions with human participants. Inclusion of studies was based solely on predefined eligibility critaria, without regard to the geographic origin of the research.
Reporting on race, ethnicity, or other socially relevant groupings	Not applicable.
Population characteristics	See above.
Recruitment	This study did not involve primary data collection.
Ethics oversight	Ethical approval was not applicable to this study, as it involved the analysis of existing data. Informed consent and participant compensation were obtained in the original studies, as reported in the respective publications.

Note that full information on the approval of the study protocol must also be provided in the manuscript.

Field-specific reporting

Please select the one below that is the best fit for your research. If you are not sure, read the appropriate sections before making your selection.

☐ Life sciences ☒ Behavioural & social sciences ☐ Ecological, evolutionary & environmental sciences

For a reference copy of the document with all sections, see nature.com/documents/nr-reporting-summary-flat.pdf

Behavioural & social sciences study design

All studies must disclose on these points even when the disclosure is negative.

Study description	This study is a quantitative systematic review and meta-analysis of experimental studies examining the illusory truth effect.
Research sample	The sample for this meta-analysis consisted of 31,184 participants aggregated across 182 experimental studies included in the review. Participants were predominantly adults recruited from general population samples, including university students and online survey panels, with ages typically ranging from adolescence to older adulthood as reported in the original studies. Both male and female participants were included across studies, although detailed demographics breakdowns (e.g., age distributions, sex ratios) were not consistently synthesized.<br>The sample is not representative of the global population, as most studies were conducted in Western contexts, primarily in Europe and North America.<br>This aggregated sample is appropriate for the study objectives, as the aim was to estimate the magnitude and moderators of the illusory truth effect across experimental contexts rather than to characterize population-level prevalence.
Sampling strategy	Not applicable.
Data collection	This study did not involve primary data collection. Data were collected by extracting summary statistics (e.g., effect sizes, means, standard deviations, sample sizes, and moderator variables) from dissertations, published articles, supplementary materials, and publicly available datasets when available. No human participants were directly involved in the data collection for this study, and no experimental sessions were conducted by the authors.
Timing	Data extraction was conducted from published and publicly available studies identified through systematic literature searches. The initial data collection and extraction took place between September 2022 and February 2025, corresponding to the original search and the subsequent update of the systematic review during the peer-review process.
Data exclusions	All exclusions occurred at the study and effect size selection stage and were based on pre-established inclusion and exclusion criteria defined prior to data extraction. The number of excluded studies and effect sizes, along with the rationale for each exclusion, is reported in the Methods section, Figure 4, and detailed in Supplementary Data 5.
Non-participation	Not applicable.
Randomization	Not applicable.

Reporting for specific materials, systems and methods

We require information from authors about some types of materials, experimental systems and methods used in many studies. Here, indicate whether each material, system or method listed is relevant to your study. If you are not sure if a list item applies to your research, read the appropriate section before selecting a response.

Materials & experimental systems

n/a	Involved in the study
☒	☐ Antibodies
☒	☐ Eukaryotic cell lines
☒	☐ Palaeontology and archaeology
☒	☐ Animals and other organisms
☒	☐ Clinical data
☒	☐ Dual use research of concern
☒	☐ Plants

Methods

n/a	Involved in the study
☒	☐ ChIP-seq
☒	☐ Flow cytometry
☒	☐ MRI-based neuroimaging

Plants

Seed stocks	NA
Novel plant genotypes	NA
Authentication	NA
